# Supplementary material for: National and subnational short-term forecasting of COVID-19 in Germany and Poland during early 2021
Source: Commun Med (Lond). 2022 Oct 31;2:136. doi: 10.1038/s43856-022-00191-8 (PMC9622804; doi:10.1038/s43856-022-00191-8)
Supplement: Supplementary file 3 — Supplemental Materials [file 43856_2022_191_MOESM3_ESM.pdf]

Supplementary Materials for Bracher et al (2021): National and subnational short-term forecasting of COVID-19 in Germany and Poland during early 2021

Johannes Bracher[1,2,*], Daniel Wolffram[1,2,3], Jannik Deuschel[1], Konstantin Görgen[1], Jakob L. Ketterer[1], Alexander Ullrich[4], Sam Abbott[5], Maria V. Barbarossa[6], Dimitris Bertsimas[7], Sangeeta Bhatia[8], Marcin Bodych[9], Nikos I. Bosse[5], Jan Pablo Burgard[10], Lauren Castro[11], Geoffrey Fairchild[11], Jochen Fiedler[12], Jan Fuhrmann[13], Sebastian Funk[5], Anna Gambin[14], Krzysztof Gogolewski[14], Stefan Heyder[15], Thomas Hotz[15], Yuri Kheifetz[16], Holger Kirsten[16], Tyll Krueger[9], Elena Krymova[17], Neele Leithäuser[12], Michael L. Li[18], Jan H. Meinke[19], Błażej Miasojedow[14], Isaac J. Michaud[20], Jan Mohring[12], Pierre Nouvellet[21], Jedrzej M. Nowosielski[22], Tomasz Ozanski[9], Maciej Radwan[22], Franciszek Rakowski[22], Markus Scholz[16], Saksham Soni[18], Ajitesh Srivastava[23], Tilmann Gneiting[2,24], Melanie Schienle[1,*]

August 25, 2022

[1]Chair of Statistics and Econometrics, Karlsruhe Institute of Technology (KIT), Karlsruhe, Germany
[2]Computational Statistics Group, Heidelberg Institute for Theoretical Studies (HITS), Heidelberg, Germany
[3]HIDSS4Health - Helmholtz Information and Data Science School for Health, Karlsruhe/Heidelberg, Germany
[4]Robert Koch Institute (RKI), Berlin, Germany
[5]London School of Hygiene and Tropical Medicine, London, UK
[6]Frankfurt Institute for Advanced Studies, Frankfurt, Germany
[7]Sloan School of Management, Massachusetts Institute of Technology, Cambridge MA, USA
[8]MRC Centre for Global Infectious Disease Analysis, Abdul Latif Jameel Institute for Disease and Emergency Analytics (J-IDEA), Imperial College London, London, UK
[9]Wroclaw University of Science and Technology, Wroclaw, Poland
[10]Economic and Social Statistics Department, University of Trier, Trier, Germany
[11]Information Systems and Modeling, Los Alamos National Laboratory, Los Alamos, USA
[12]Fraunhofer Institute for Industrial Mathematics (ITWM), Kaiserslautern, Germany
[13]Institute for Applied Mathematics, University of Heidelberg, Heidelberg, Germany
[14]Faculty of Mathematics, Informatics, and Mechanics, University of Warsaw, Warsaw, Poland
[15]Institute of Mathematics, Technische Universität Ilmenau, Ilmenau, Germany
[16]Institute for Medical Informatics, Statistics and Epidemiology, University of Leipzig, Leipzig, Germany
[17]Swiss Data Science Center, ETH Zürich and EPF Lausanne, Zürich, Switzerland
[18]Operations Research Center, Massachusetts Institute of Technology, Cambridge, MA, USA
[19]Jülich Supercomputing Centre, Forschungszentrum Jülich, Jülich, Germany
[20]Statistical Sciences Group, Los Alamos National Laboratory, Los Alamos, USA
[21]School of Life Sciences, University of Sussex, Brighton, UK
[22]Interdisciplinary Centre for Mathematical and Computational Modelling, University of Warsaw, Warsaw, Poland
[23]Ming Hsieh Department of Computer and Electrical Engineering, University of Southern California, Los Angeles, USA
[24]Institute for Stochastics, Karlsruhe Institute of Technology (KIT), Karlsruhe, Germany

S1 Detailed description of new models

We only provide detailed descriptions of models which were added to our project for the second evaluation period. Descriptions for the other models can be found in Supplementary Note 3 of Bracher et al (2021). A more detailed documentation of the `LeipzigIMISE-SECIR` and `SDSC_ISG-TrendModel` models which had not been available at the appearance of Bracher (2021) can be found in Kheifetz et al (2021) and Krymova et al (2021), respectively.

itwm-dSEIR Fraunhofer-ITWM's predictions are based on a cohort model that groups people according to four age groups and according to the status infected, detected and since 19 April successfully vaccinated (i.e., this extension was added after the evaluation period). The dynamics of the epidemic are described by integral equations, assuming an infectious period with fixed onset, end and infectivity. The most important parameters are contact rates between age groups, detection rates and times, and death rates and times, which are adjusted to the historical data of the RKI. For forecasts, the simulation is continued with the parameters determined for the last week. In principle, the forecast quality could be improved by anticipating the effects of events such as the end of public holidays on contact and detection rates. However, this is not yet done in the automatic submissions. All calculations use automatic differentiation. This speeds up parameter adjustment and allows for error estimates. The latter are determined by comparing counted and simulated cases and by matching the empirical standard deviations with the standard deviations predicted by the calculated sensitivities. The model is described in detail in https://www.itwm.fraunhofer.de/de/presse-publikationen/presseinformationen/2021/2021-06-22_Dritte_Welle_Starker-Effekt-von-Schnelltests-an-Schulen.html.

Karlen-pypm The python Population Modeller (pyPM, Karlen 2020) is a mechanistic modeling framework to describe viral spread via discrete-time difference equations. In a pyPM model, different population objects are connected by a list of directional connector objects. The adjustable parameters of the model are stored in parameter objects. The core of the model consists of a model of the infection cycle involving the susceptible, infected (but not yet contagious) and contagious parts of the population. The contagious population is modelled in more detail by introducing symptomatic, test-positive, hospitalized (normal ward and ICU) and deceased populations. The model takes time series of cases, deaths and intensive care occupancy as data inputs. Forecasts are generated at the regional level (German states) first and subsequently aggregated to the national level. Starting from 1 March 2021, the model was stratified into spread of the wild type of the virus and the B.1.1.7 variant, and integrated genetic sequencing data on their respective importance.

S2 Additional forecast visualizations

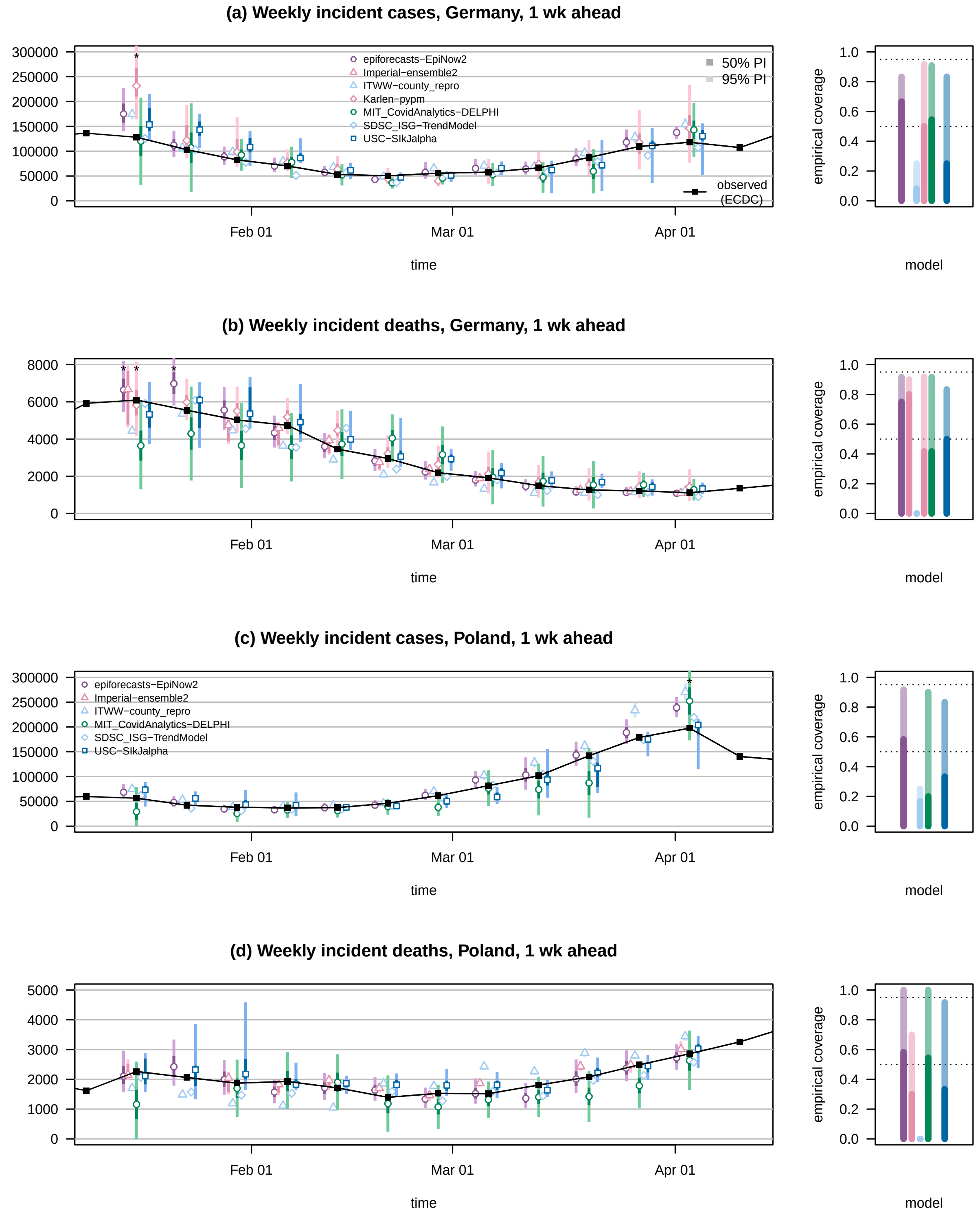

Figure S1: One-week-ahead forecasts of confirmed cases and deaths from COVID-19 in Germany and Poland. Asterisks mark prediction intervals exceeding the upper plot limit. The figure shows forecasts from models not displayed in Figure 2. Colored points represent predictive medians, dark and light bars show 50% and 95% prediction intervals, respectively. Asterisks mark prediction intervals exceeding the upper plot limit.

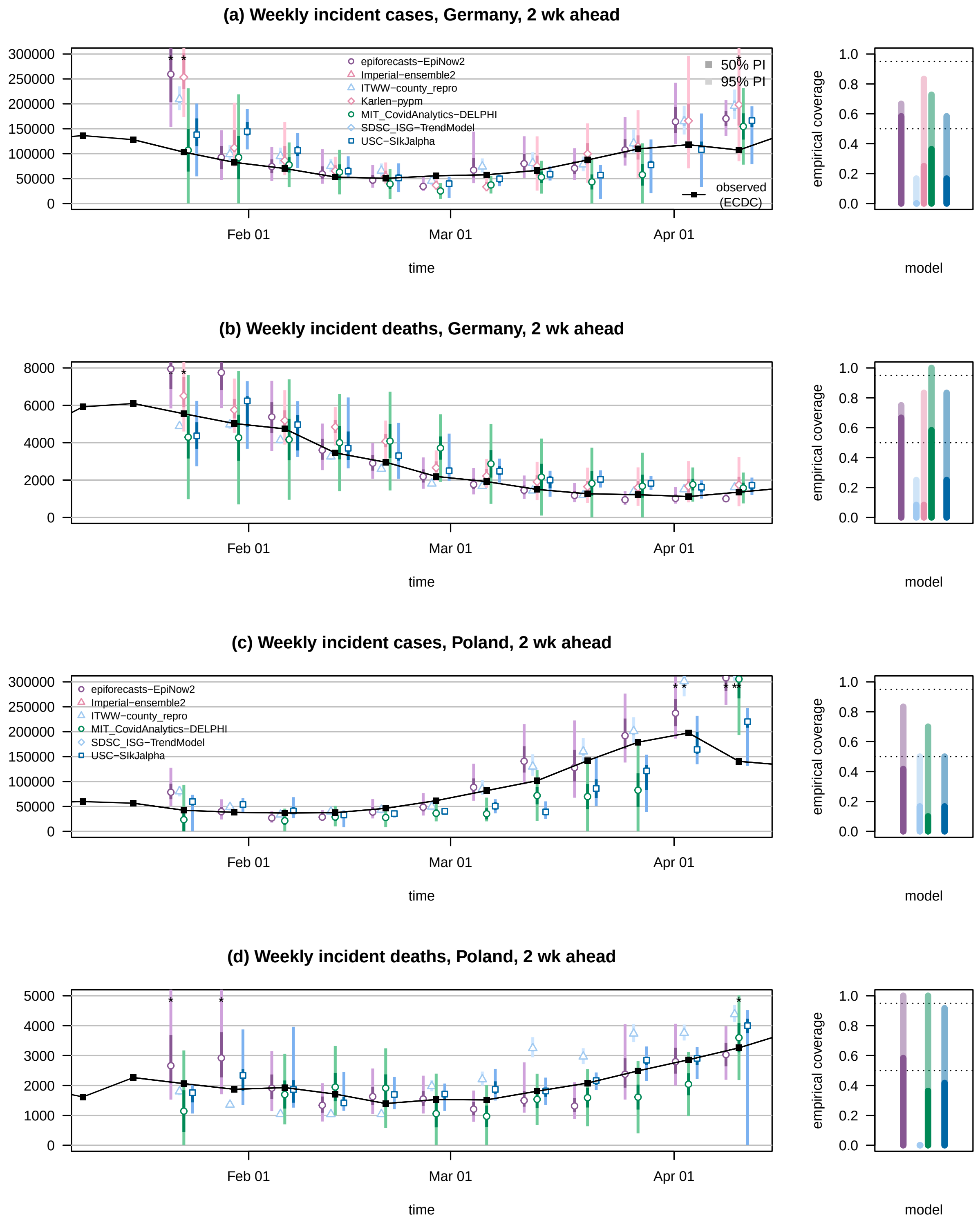

Figure S2: Two-week-ahead forecasts of confirmed cases and deaths from COVID-19 in Germany and Poland, same models as displayed in Figure 3. Colored points represent predictive medians, dark and light bars show 50% and 95% prediction intervals, respectively. Asterisks mark prediction intervals exceeding the upper plot limit.

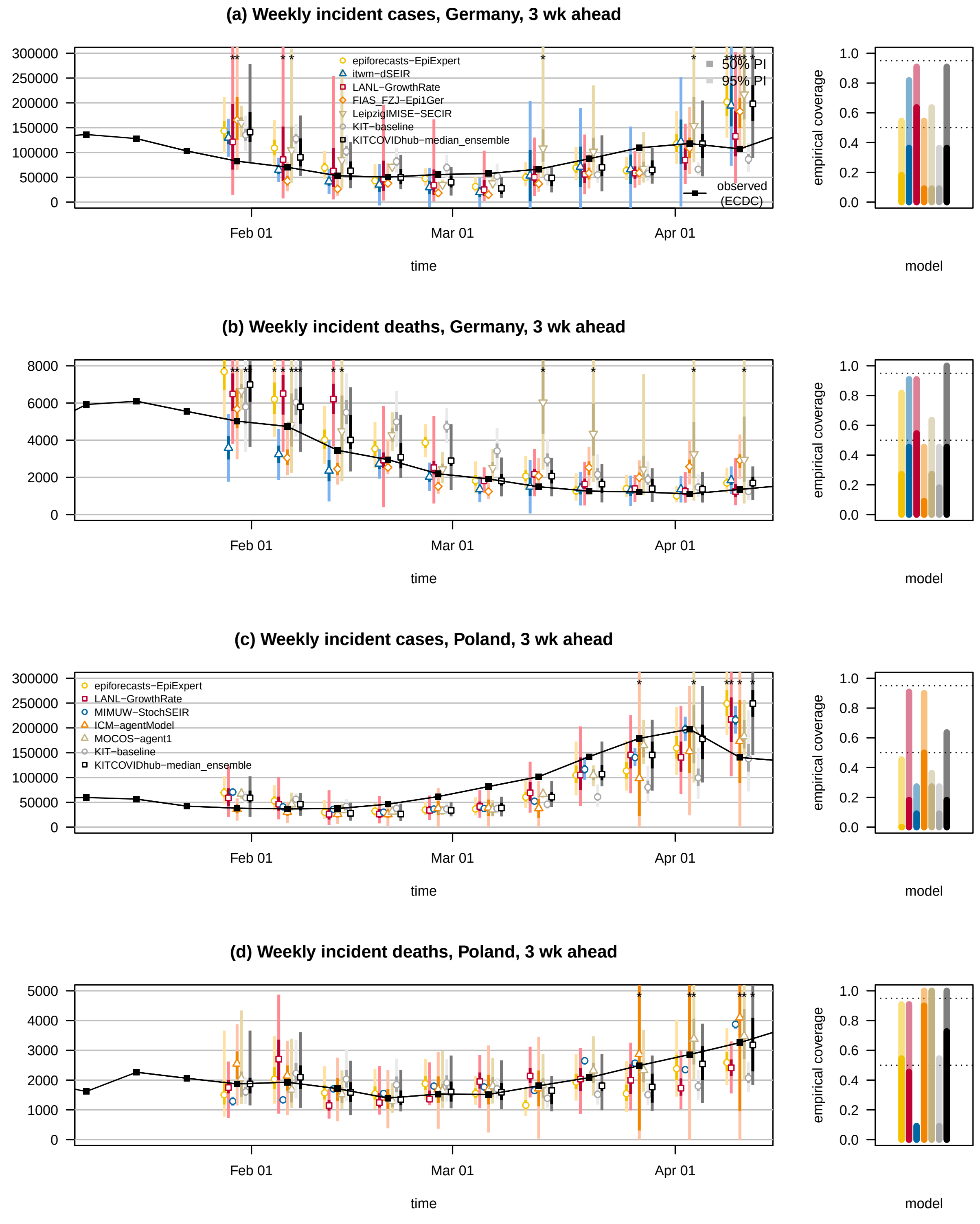

Figure S3: Three-week-ahead forecasts of confirmed cases and deaths from COVID-19 in Germany and Poland, same models as displayed in Figure 3. Colored points represent predictive medians, dark and light bars show 50% and 95% prediction intervals, respectively. Asterisks mark prediction intervals exceeding the upper plot limit.

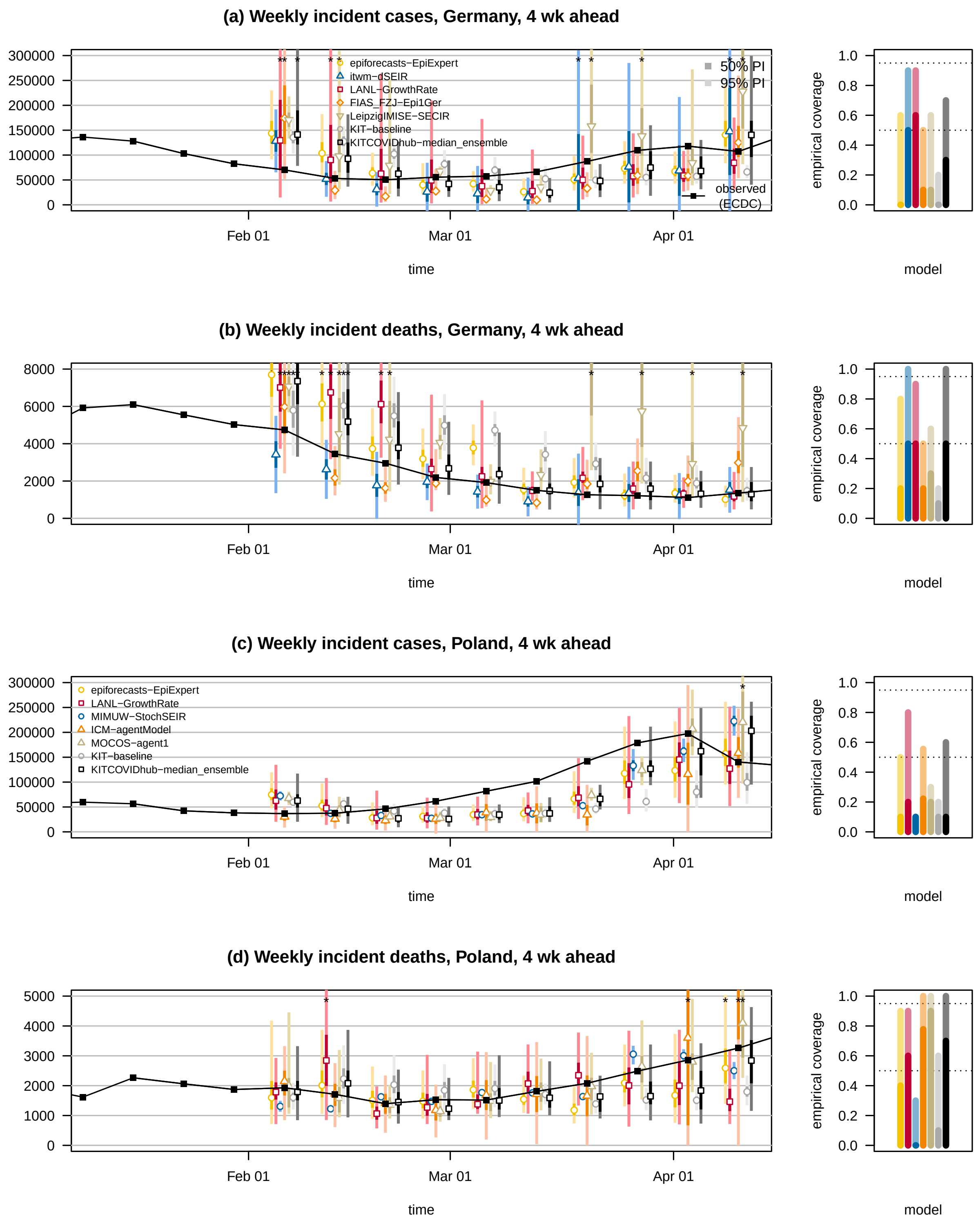

Figure S4: Four-week-ahead forecasts of confirmed cases and deaths from COVID-19 in Germany and Poland, same models as displayed in Figure 3. Colored points represent predictive medians, dark and light bars show 50% and 95% prediction intervals, respectively. Asterisks mark prediction intervals exceeding the upper plot limit.

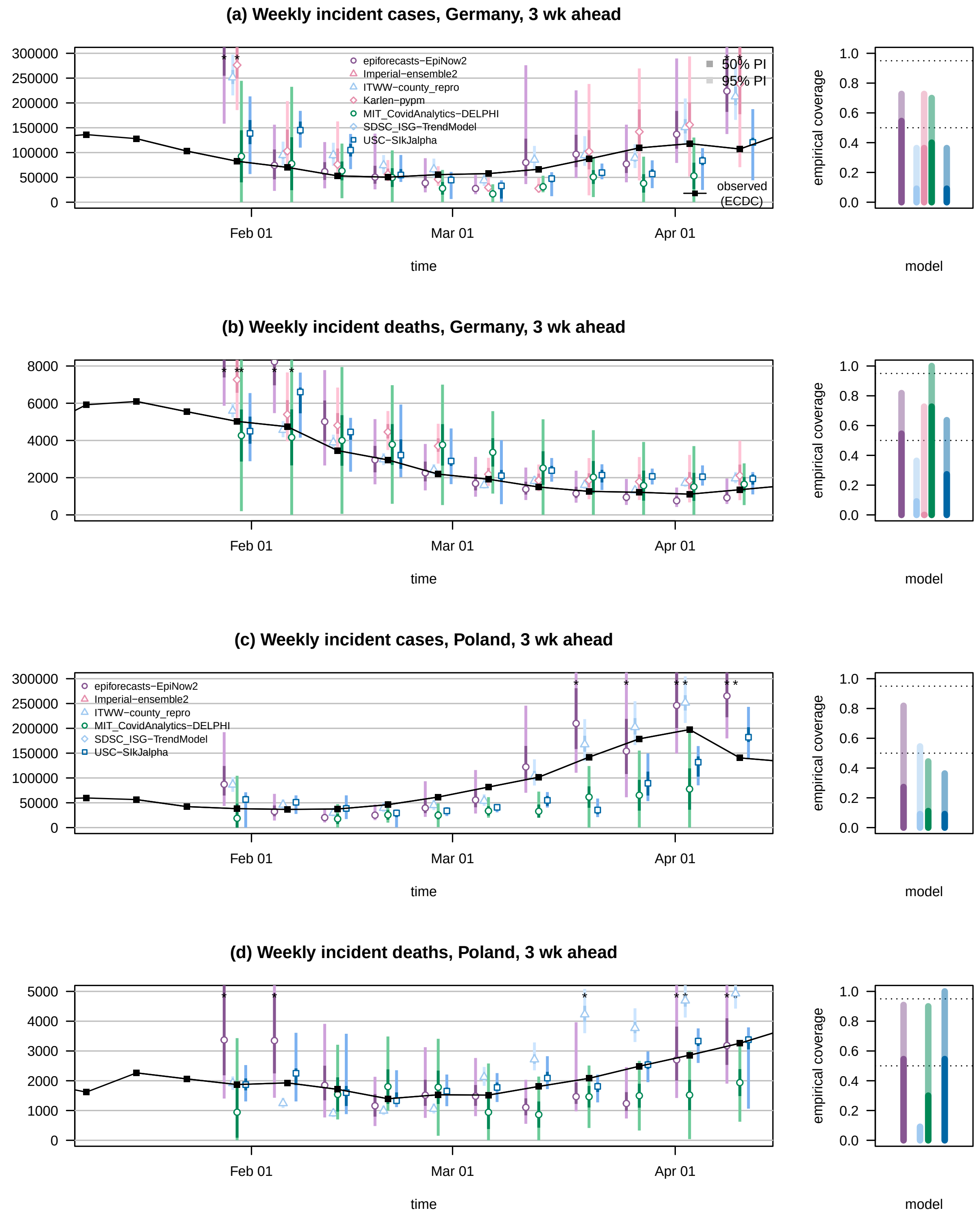

Figure S5: Three-week-ahead forecasts of confirmed cases and deaths from COVID-19 in Germany and Poland, same models as displayed in Figure S1. Colored points represent predictive medians, dark and light bars show 50% and 95% prediction intervals, respectively. Asterisks mark prediction intervals exceeding the upper plot limit.

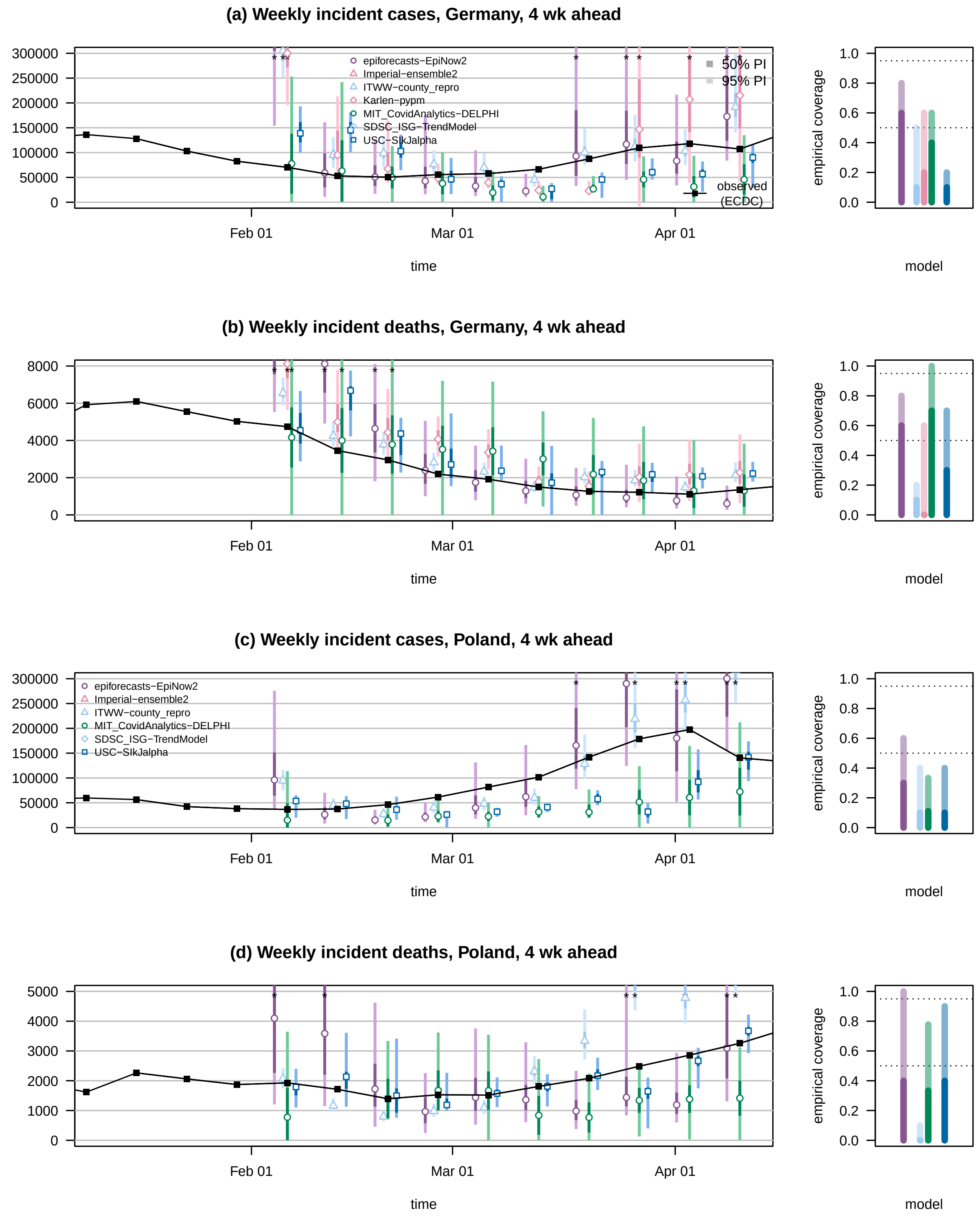

Figure S6: Four-week-ahead forecasts of confirmed cases and deaths from COVID-19 in Germany and Poland, same models as displayed in Figure S1. Colored points represent predictive medians, dark and light bars show 50% and 95% prediction intervals, respectively. Asterisks mark prediction intervals exceeding the upper plot limit.

S3 Decomposition of average WIS values

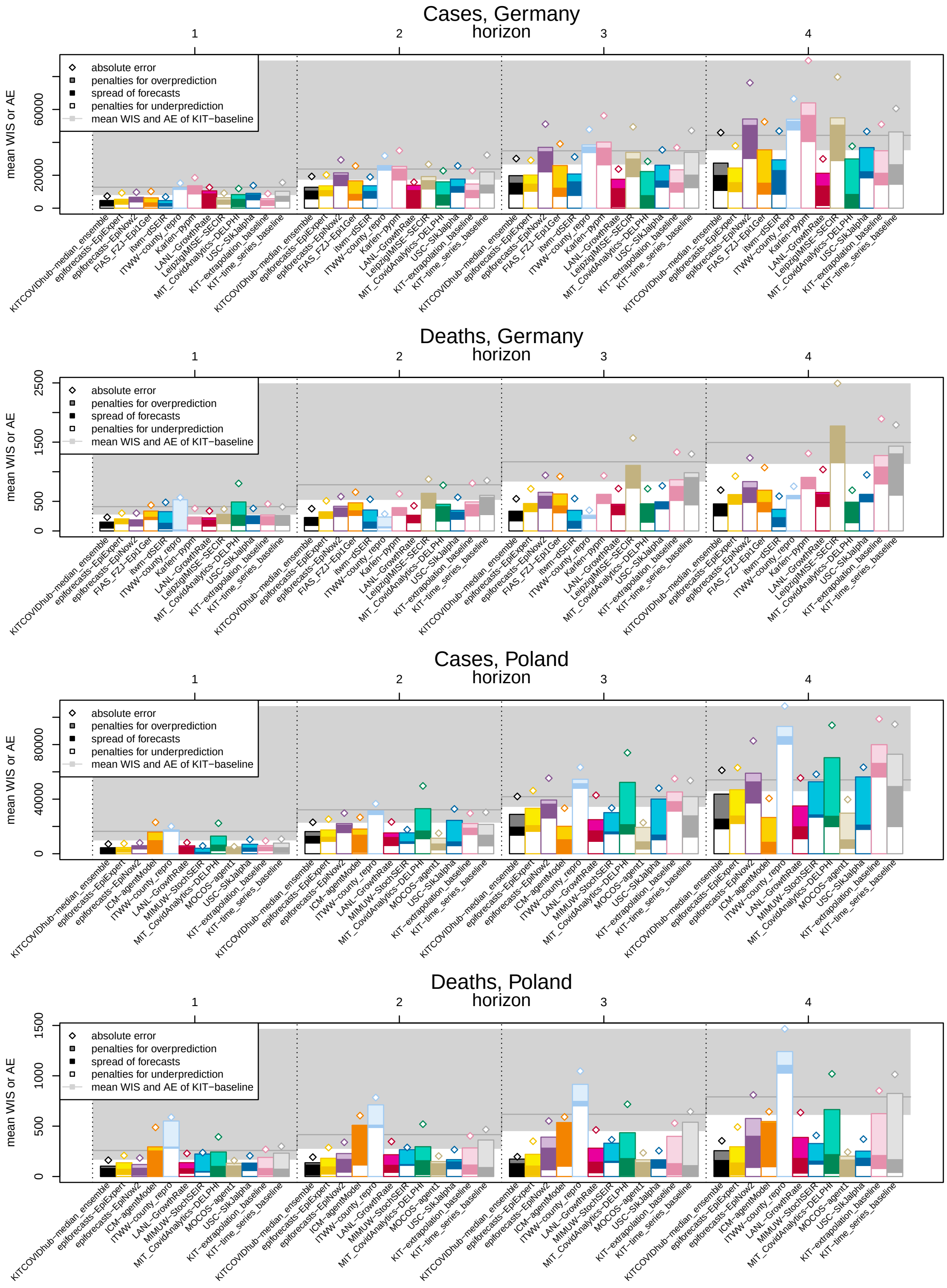

Figure S7: Average weighted interval score and absolute error achieved by models across countries, targets and forecast horizons. The grey area represents the performance of the baseline model `KIT-baseline`. WIS values are decomposed into components for forecast spread, overprediction and underprediction.

S4 Individual WIS values

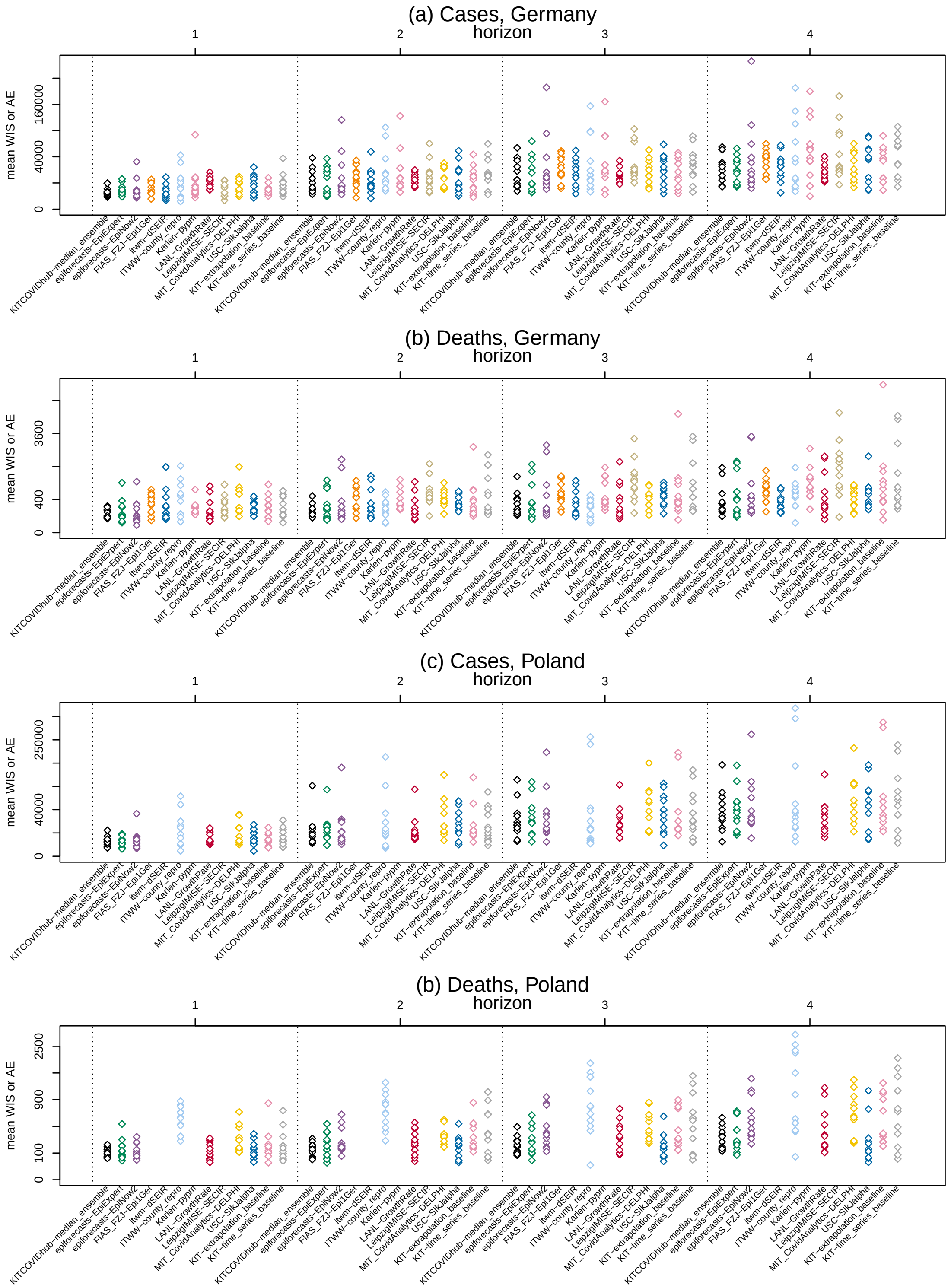

Figure S8: Individual weighted interval scores achieved by models across countries, targets and forecast horizons. Each dot represents one score achieved over the 12-week evaluation period.

S5 Weights in inverse-WIS ensembles

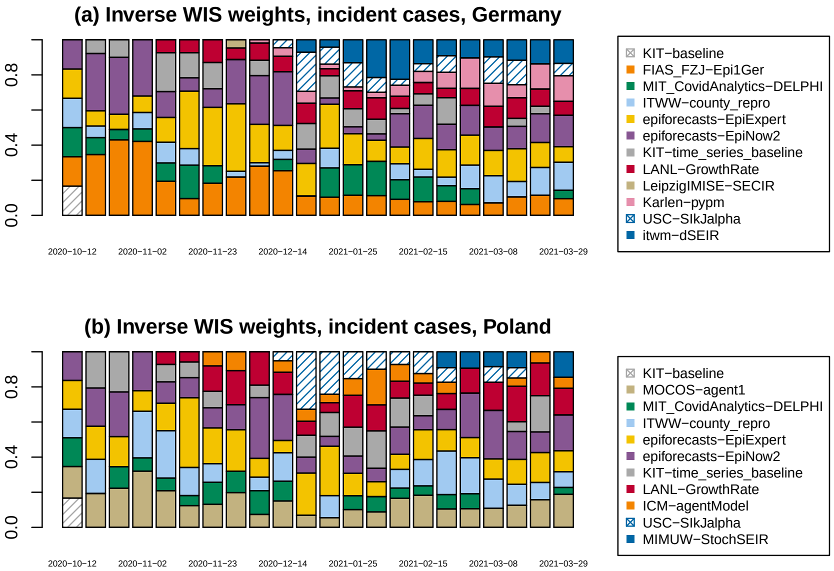

Figure S9: Weights in KITCOVIDhub-inverse_wis_ensemble for incident cases in Germany and Poland, October 2020–March 2021 (i.e., combined for the study periods of Bracher et al (2021) and the present manuscript).

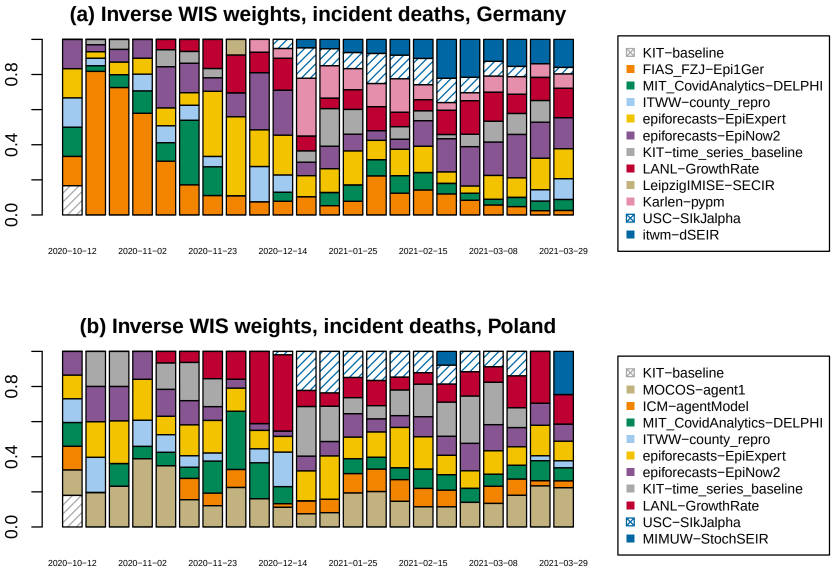

Figure S10: Weights in KITCOVIDhub-inverse_wis_ensemble for incident deaths in Germany and Poland, October 2020–March 2021 (i.e., combined for the study periods of Bracher et al (2021) and the present manuscript).

S6 Visualization of behaviour at turning points

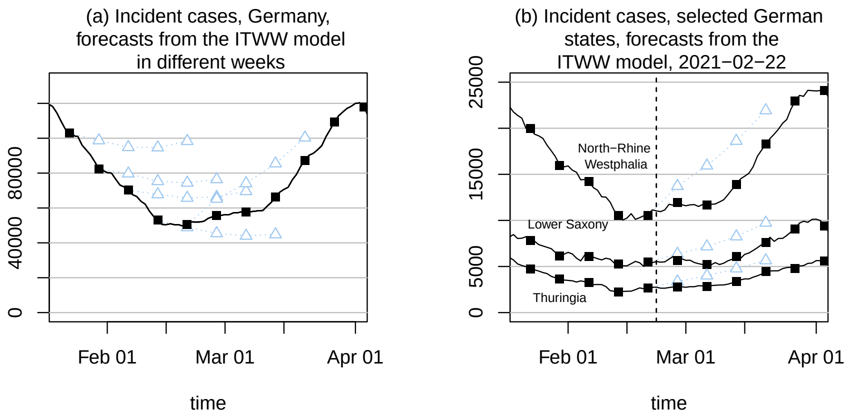

Figure S11: **a** Forecasts of cases in Germany by the `ITWW-county_repro` model, 25 January to 22 February 2021. **b** Forecasts for cases in selected German states by the `ITWW-county_repro` model, 22 February 2021.

S7 Additional summary tables on forecast evaluation

Table S1: Forecast evaluation for Germany and Poland in terms of relative AE and WIS, 1–4 weeks ahead. The relative values are obtained by dividing the mean absolute error or WIS of a given model by the respective value achieved by the baseline model. Values below 1 indicate better performance than the baseline, values above worse performance.

	**Germany**															
	1 wk case		2 wk case		3 wk case		4 wk case		1 wk death		2 wk death		3 wk death		4 wk death	
Model	relAE	relWIS	relAE	relWIS	relAE	relWIS	relAE	relWIS	relAE	relWIS	relAE	relWIS	relAE	relWIS	relAE	relWIS
epiforecasts-EpiExpert	0.73	0.68	0.85	0.79	0.83	0.76	0.86	0.69	0.73	0.74	0.65	0.61	0.61	0.56	0.62	0.54
epiforecasts-EpiNow2	0.76	0.84	1.23	1.24	1.47	1.39	1.72	1.54	0.73	0.68	0.74	0.79	0.80	0.78	0.83	0.74
FIAS_FZJ-Epi1Ger	0.80	0.79	1.08	0.96	1.12	0.98	1.19	1.01	1.06	1.21	0.84	0.91	0.79	0.75	0.72	0.61
IHME-CurveFit									1.26		0.84		0.72		0.65	
Imperial-ensemble2									0.47	0.49						
itwm-dSEIR	0.54	0.58	0.80	0.79	0.89	0.78	1.06	0.83	1.18	1.18	0.68	0.67	0.47	0.42	0.39	0.32
ITWW-county_repro	1.19	1.56	1.34	1.49	1.37	1.45	1.50	1.53	1.37	1.91	0.37	0.45	0.30	0.32	0.50	0.53
Karlen-pypm	1.45	1.71	1.47	1.46	1.61	1.51	2.03	1.82	0.93	0.84	0.80	0.75	0.80	0.74	0.88	0.80
LANL-GrowthRate	0.99	1.33	0.66	0.80	0.68	0.66	0.68	0.60	0.82	0.80	0.54	0.51	0.61	0.54	0.69	0.57
LeipzigIMISE-SECIR	0.72	0.80	1.12	1.11	1.42	1.28	1.80	1.56	0.90	1.01	1.12	1.21	1.34	1.33	1.67	1.57
MIT_CovidAnalytics-DELPHI	0.93	1.04	0.96	0.92	0.81	0.84	0.85	0.85	1.95	1.77	0.99	0.86	0.61	0.55	0.46	0.43
SDSC_ISG-TrendModel	0.62								1.06							
USC-SIkJalpha	1.08	1.13	1.08	1.02	1.02	0.98	1.05	1.05	0.93	0.92	0.73	0.66	0.65	0.59	0.63	0.54
KIT-baseline	1.00	1.00	1.00	1.00	1.00	1.00	1.00	1.00	1.00	1.00	1.00	1.00	1.00	1.00	1.00	1.00
KIT-extrapolation_baseline	0.69	0.72	0.96	0.85	1.06	0.88	1.15	0.99	1.11	0.97	1.03	0.93	1.14	1.04	1.27	1.12
KIT-time_series_baseline	1.22	1.29	1.36	1.27	1.35	1.28	1.37	1.32	0.99	0.95	1.09	1.15	1.11	1.18	1.20	1.26
KITCOVIDhub-inverse_wis_ensemble	0.67	0.67	0.92	0.80	0.98	0.81	1.11	0.86	0.53	0.54	0.39	0.39	0.42	0.38	0.45	0.40
KITCOVIDhub-mean_ensemble	0.66	0.66	0.92	0.79	0.97	0.80	1.10	0.84	0.53	0.55	0.44	0.42	0.44	0.38	0.48	0.38
KITCOVIDhub-median_ensemble	0.58	0.59	0.81	0.73	0.86	0.74	1.04	0.78	0.56	0.54	0.48	0.43	0.46	0.40	0.46	0.40
	**Poland**															
	1 wk case		2 wk case		3 wk case		4 wk case		1 wk death		2 wk death		3 wk death		4 wk death	
Model	relAE	relWIS	relAE	relWIS	relAE	relWIS	relAE	relWIS	relAE	relWIS	relAE	relWIS	relAE	relWIS	relAE	relWIS
epiforecasts-EpiExpert	0.46	0.47	0.79	0.77	1.11	0.97	1.16	1.02	0.81	0.82	0.69	0.66	0.57	0.49	0.62	0.48
epiforecasts-EpiNow2	0.48	0.61	0.92	0.97	1.32	1.15	1.53	1.29	0.71	0.71	0.82	0.83	0.89	0.87	1.02	0.94
ICM-agentModel	1.40	1.63	0.83	0.80	0.80	0.59	0.75	0.58	1.89	1.77	1.46	1.84	0.96	1.19	0.81	0.89
IHME-CurveFit									1.45		1.25		1.03		0.64	
Imperial-ensemble2									0.73	0.83						
ITWW-county_repro	1.22	1.78	1.14	1.38	1.51	1.59	2.00	2.04	2.28	3.31	1.89	2.59	1.69	2.03	1.85	2.03
LANL-GrowthRate	0.50	0.59	0.72	0.67	1.03	0.73	1.03	0.76	0.89	0.82	0.83	0.79	0.75	0.62	0.80	0.63
MIMUW-StochSEIR	0.35	0.41	0.55	0.68	0.80	0.88	1.08	1.15	0.92	1.34	0.69	0.97	0.59	0.73	0.52	0.54
MIT_CovidAnalytics-DELPHI	1.36	1.33	1.54	1.45	1.77	1.52	1.74	1.54	1.52	1.46	1.25	1.08	1.16	0.97	1.29	1.09
MOCOS-agent1	0.32	0.51	0.47	0.50	0.54	0.56	0.73	0.65	0.61	0.79	0.49	0.54	0.38	0.37	0.31	0.33
SDSC_ISG-TrendModel	0.39								1.02							
USC-SIkJalpha	0.63	0.71	1.02	1.08	1.15	1.17	1.17	1.23	0.80	0.80	0.64	0.61	0.41	0.37	0.47	0.41
KIT-baseline	1.00	1.00	1.00	1.00	1.00	1.00	1.00	1.00	1.00	1.00	1.00	1.00	1.00	1.00	1.00	1.00
KIT-extrapolation_baseline	0.58	0.62	0.92	0.98	1.32	1.32	1.82	1.74	1.04	1.14	0.97	1.03	0.86	0.89	1.08	1.02
KIT-time_series_baseline	0.66	0.80	0.94	0.95	1.28	1.22	1.75	1.59	1.16	1.39	1.12	1.32	1.04	1.20	1.28	1.35
KITCOVIDhub-inverse_wis_ensemble	0.45	0.48	0.73	0.69	0.92	0.79	1.10	0.93	0.58	0.67	0.47	0.52	0.39	0.39	0.52	0.43
KITCOVIDhub-mean_ensemble	0.42	0.49	0.74	0.69	0.99	0.80	1.16	0.96	0.55	0.69	0.42	0.55	0.27	0.41	0.45	0.43
KITCOVIDhub-median_ensemble	0.43	0.45	0.72	0.72	1.00	0.84	1.13	0.95	0.63	0.62	0.46	0.50	0.32	0.38	0.45	0.42

Table S2: Forecast evaluation for Germany and Poland, 3 and 4 weeks ahead (incidence scale, based on RKI/MZ data). $C_{0.5}$ and $C_{0.95}$ denote coverage rates of the 50% and 95% prediction intervals; AE and WIS stand for the mean absolute error and mean weighted interval score.

	**Germany**															
	3 wk ahead case				4 wk ahead case				3 wk ahead death				4 wk ahead death			
Model	AE	WIS	$C_{0.5}$	$C_{0.95}$	AE	WIS	$C_{0.5}$	$C_{0.95}$	AE	WIS	$C_{0.5}$	$C_{0.95}$	AE	WIS	$C_{0.5}$	$C_{0.95}$
epiforecasts-EpiExpert	29,111	20,140	0.25	0.58	37,879	24,429	0.08	0.67	712	463	0.25	0.83	925	614	0.25	0.83
epiforecasts-EpiNow2	51,095	36,966	0.50	0.75	76,218	54,216	0.50	0.67	940	653	0.50	0.75	1,234	834	0.50	0.75
FIAS_FZJ-Epi1Ger	39,102	25,939	0.08	0.58	52,516	35,472	0.08	0.58	920	625	0.08	0.50	1,071	688	0.17	0.50
IHME-CurveFit									839				967			
Imperial-ensemble2																
itwm-dSEIR	31,177	20,772	0.33	0.83	46,895	29,388	0.42	0.92	547	348	0.42	0.92	588	366	0.42	1.00
ITWW-county_repro	47,796	38,477	0.08	0.33	66,520	54,070	0.08	0.42	350	266	0.08	0.33	753	599	0.08	0.17
Karlen-pypm	56,242	40,195	0.33	0.75	89,745	63,976	0.17	0.67	933	618	0.00	0.75	1,309	905	0.00	0.67
LANL-GrowthRate	23,760	17,577	0.67	0.92	29,959	21,224	0.67	0.92	715	450	0.50	0.92	1,037	651	0.50	0.92
LeipzigIMISE-SECIR	49,422	33,952	0.08	0.67	79,741	54,914	0.08	0.67	1,570	1,107	0.25	0.67	2,493	1,773	0.25	0.67
MIT_CovidAnalytics-DELPHI	*28,404	*22,268	0.45	0.73	*37,661	*29,934	0.45	0.64	713	459	0.75	1.00	688	486	0.75	1.00
SDSC_ISG-TrendModel																
USC-SIkJalpha	35,434	26,133	0.08	0.42	46,612	36,819	0.17	0.25	762	492	0.25	0.67	947	617	0.25	0.67
KIT-baseline	34,871	26,554	0.08	0.42	44,270	35,228	0.00	0.25	1,168	834	0.17	0.50	1,494	1,132	0.08	0.33
KIT-extrapolation_baseline	36,817	23,333	0.17	0.83	50,965	34,906	0.17	0.50	1,331	865	0.25	0.75	1,893	1,273	0.17	0.75
KIT-time_series_baseline	47,164	33,930	0.17	0.58	60,577	46,342	0.33	0.58	1,299	983	0.50	0.75	1,791	1,432	0.42	0.83
KITCOVIDhub-inverse_wis_ensemble	34,038	21,456	0.33	0.92	48,943	30,201	0.42	0.83	494	320	0.75	1.00	678	450	0.58	1.00
KITCOVIDhub-mean_ensemble	33,754	21,288	0.25	0.83	48,688	29,683	0.33	0.83	518	318	0.50	1.00	712	435	0.50	1.00
KITCOVIDhub-median_ensemble	30,154	19,767	0.33	0.92	45,928	27,397	0.25	0.75	543	332	0.42	1.00	690	456	0.42	1.00
	**Poland**															
	3 wk ahead case				4 wk ahead case				3 wk ahead death				4 wk ahead death			
Model	AE	WIS	$C_{0.5}$	$C_{0.95}$	AE	WIS	$C_{0.5}$	$C_{0.95}$	AE	WIS	$C_{0.5}$	$C_{0.95}$	AE	WIS	$C_{0.5}$	$C_{0.95}$
epiforecasts-EpiExpert	46,220	33,301	0.00	0.42	63,055	46,882	0.08	0.42	350	220	0.58	0.92	491	295	0.42	0.92
epiforecasts-EpiNow2	55,368	39,353	0.25	0.75	82,709	59,002	0.25	0.50	552	391	0.58	0.92	810	576	0.50	1.00
ICM-agentModel	*33,401	*20,103	0.55	0.91	* 40,524	*26,560	0.36	0.64	* 592	*536	0.91	1.00	* 643	* 546	0.82	1.00
IHME-CurveFit									637				505			
Imperial-ensemble2																
ITWW-county_repro	63,327	54,555	0.08	0.50	108,188	93,286	0.08	0.33	1,047	915	0.08	0.08	1,466	1,241	0.00	0.08
LANL-GrowthRate	42,864	24,958	0.17	0.83	55,486	35,013	0.17	0.75	463	281	0.42	0.92	635	388	0.58	0.83
MIMUW-StochSEIR	33,532	30,118	0.08	0.25	58,203	52,730	0.08	0.08	365	331	0.08	0.08	408	327	0.08	0.33
MIT_CovidAnalytics-DELPHI	*74,004	*52,292	0.10	0.40	* 94,153	*70,385	0.10	0.30	* 717	*435	0.36	0.91	*1,019	* 665	0.27	0.73
MOCOS-agent1	22,746	19,350	0.33	0.42	39,711	29,751	0.25	0.42	235	166	0.92	1.00	243	202	0.92	1.00
SDSC_ISG-TrendModel																
USC-SIkJalpha	48,043	40,067	0.08	0.33	63,352	56,280	0.08	0.33	256	166	0.50	1.00	371	252	0.42	0.92
KIT-baseline	41,804	34,355	0.08	0.33	54,127	45,833	0.17	0.25	618	451	0.08	0.50	791	611	0.08	0.50
KIT-extrapolation_baseline	55,011	45,278	0.33	0.42	98,777	79,912	0.17	0.33	530	399	0.58	0.67	852	624	0.42	0.58
KIT-time_series_baseline	53,619	41,830	0.42	0.67	94,873	72,907	0.33	0.67	644	539	0.42	0.58	1,014	824	0.33	0.50
KITCOVIDhub-inverse_wis_ensemble	38,590	27,016	0.25	0.67	59,449	42,678	0.25	0.58	242	178	0.75	1.00	408	265	0.67	1.00
KITCOVIDhub-mean_ensemble	41,216	27,591	0.25	0.67	62,918	43,800	0.25	0.50	169	185	0.83	1.00	353	263	0.75	1.00
KITCOVIDhub-median_ensemble	41,979	28,852	0.17	0.58	61,177	43,683	0.08	0.58	196	173	0.75	1.00	354	256	0.75	1.00

*Asterisks mark entries where scores were imputed for at least one week. Weighted interval scores and absolute errors were imputed with the worst (largest) score achieved by any other forecast for the respective target and week. Models marked thus received a pessimistic assessment of their performance. If a model covered less than two thirds of the evaluation period, results are omitted.

Table S3: Forecast evaluation for Germany and Poland, 1 and 2 weeks ahead (cumulative scale, based on RKI/MZ data). $C_{0.5}$ and $C_{0.95}$ denote coverage rates of the 50% and 95% prediction intervals; AE and WIS stand for the mean absolute error and mean weighted interval score.

Germany

	1 wk ahead cumul case				2 wk ahead cumul case				1 wk ahead cumul death				2 wk ahead cumul death			
Model	AE	WIS	$C_{0.5}$	$C_{0.95}$	AE	WIS	$C_{0.5}$	$C_{0.95}$	AE	WIS	$C_{0.5}$	$C_{0.95}$	AE	WIS	$C_{0.5}$	$C_{0.95}$
epiforecasts-EpiExpert	9,252	5,415	0.25	1.00	29,303	18,479	0.25	0.75	300	204	0.50	0.92	739	496	0.42	0.92
epiforecasts-EpiNow2	14,097	11,023	0.67	0.75	42,806	32,126	0.58	0.75	588	457	0.67	0.83	1,130	804	0.58	0.83
FIAS_FZJ-Epi1Ger	10,859	6,690	0.50	0.92	35,351	21,697	0.17	0.83	578	458	0.00	0.25	976	739	0.25	0.50
Imperial-ensemble2									* 193	* 136	0.80	0.90				
itwm-dSEIR	7,517	5,379	0.42	0.67	26,189	19,526	0.33	0.67	726	548	0.25	0.50	1,149	972	0.33	0.50
ITWW-county_repro	15,223	12,418	0.08	0.25	46,473	37,889	0.08	0.33	564	527	0.00	0.00	813	742	0.08	0.17
Karlen-pypm	18,532	13,629	0.50	0.92	53,323	38,243	0.33	0.92	380	232	0.42	0.92	963	596	0.42	0.92
LANL-GrowthRate	6,889	9,267	1.00	1.00	21,087	22,068	0.83	1.00	466	284	0.25	1.00	866	532	0.42	1.00
LeipzigIMISE-SECIR	17,708	12,470	0.00	0.42	41,912	29,244	0.25	0.50	1,474	1,335	0.50	0.50	3,142	2,544	0.50	0.58
MIT_CovidAnalytics-DELPHI									851	504	0.33	0.92	1,533	969	0.08	0.75
SDSC_ISG-TrendModel	10,394								384							
USC-SIkJalpha	16,854	11,177	0.33	0.83	40,016	30,407	0.17	0.58	467	314	0.33	0.67	1,035	686	0.17	0.42
KIT-baseline	12,756	7,953	0.42	0.92	35,996	26,510	0.17	0.42	411	277	0.58	0.92	1,164	811	0.25	0.75
KIT-extrapolation_baseline	8,823	5,715	0.50	1.00	31,598	19,803	0.42	0.75	456	269	0.33	1.00	1,219	748	0.33	1.00
KIT-time_series_baseline	15,583	10,281	0.25	0.75	47,712	33,183	0.25	0.67	406	263	0.67	1.00	1,247	849	0.58	1.00
KITCOVIDhub-inverse_wis_ensemble	10,649	6,614	0.58	0.92	32,451	20,251	0.33	0.83	303	183	0.50	1.00	463	319	0.67	1.00
KITCOVIDhub-mean_ensemble	9,715	6,000	0.67	1.00	31,185	19,363	0.50	1.00	280	178	0.42	1.00	451	325	0.50	1.00
KITCOVIDhub-median_ensemble	8,118	5,344	0.50	1.00	27,596	18,028	0.42	0.92	283	161	0.50	1.00	544	342	0.50	1.00

Poland

	1 wk ahead cumul case				2 wk ahead cumul case				1 wk ahead cumul death				2 wk ahead cumul death			
Model	AE	WIS	$C_{0.5}$	$C_{0.95}$	AE	WIS	$C_{0.5}$	$C_{0.95}$	AE	WIS	$C_{0.5}$	$C_{0.95}$	AE	WIS	$C_{0.5}$	$C_{0.95}$
epiforecasts-EpiExpert	21,893	16,543	0.33	0.75	36,184	24,110	0.33	0.58	316	202	0.17	0.75	499	301	0.50	1.00
epiforecasts-EpiNow2	13,372	9,851	0.42	0.83	43,134	30,915	0.42	0.75	332	261	0.58	0.83	639	440	0.58	0.83
ICM-agentModel	*14,264	*12,390	0.64	1.00	*40,403	*30,044	0.64	1.00	*279	*279	0.91	1.00	* 624	* 735	1.00	1.00
Imperial-ensemble2									*188	*138	0.30	0.70				
ITWW-county_repro	20,054	17,364	0.17	0.25	56,144	48,119	0.17	0.42	589	551	0.00	0.00	1,374	1,276	0.00	0.00
LANL-GrowthRate	8,129	5,787	0.83	1.00	30,850	19,842	0.58	1.00	229	137	0.17	0.83	573	348	0.33	0.92
MIMUW-StochSEIR	5,933	4,132	0.33	0.83	23,176	19,204	0.08	0.25	251	238	0.17	0.25	524	497	0.00	0.00
MIT_CovidAnalytics-DELPHI									*320	*199	0.55	1.00	* 823	* 513	0.27	0.82
MOCOS-agent1	5,173	4,978	0.42	0.67	19,929	15,373	0.25	0.75	158	132	0.75	1.00	349	275	0.83	1.00
SDSC_ISG-TrendModel	12,372								201							
USC-SIkJalpha	10,405	6,919	0.33	0.83	42,376	32,924	0.08	0.42	206	133	0.33	0.92	441	279	0.17	0.75
KIT-baseline	16,407	9,736	0.42	0.83	44,384	32,767	0.17	0.42	258	167	0.42	0.92	664	457	0.17	0.50
KIT-extrapolation_baseline	9,448	5,992	0.50	0.92	38,410	27,616	0.25	0.75	269	190	0.58	0.83	672	466	0.50	0.83
KIT-time_series_baseline	10,784	7,787	0.75	0.83	41,180	29,143	0.42	0.75	300	232	0.67	0.67	735	593	0.50	0.67
KITCOVIDhub-inverse_wis_ensemble	8,743	5,630	0.42	0.92	28,915	18,765	0.42	0.83	131	105	0.83	1.00	320	231	0.67	1.00
KITCOVIDhub-mean_ensemble	8,448	5,465	0.67	0.92	28,569	18,187	0.42	0.83	125	108	0.83	1.00	274	235	1.00	1.00
KITCOVIDhub-median_ensemble	6,334	4,402	0.67	1.00	28,772	19,818	0.33	0.83	144	97	0.75	1.00	280	207	0.75	1.00

*Asterisks mark entries where scores were imputed for at least one week. Weighted interval scores and absolute errors were imputed with the worst (largest) score achieved by any other forecast for the respective target and week. Models marked thus received a pessimistic assessment of their performance. If a model covered less than two thirds of the evaluation period, results are omitted.

Table S4: Forecast evaluation for Germany and Poland, 3 and 4 weeks ahead (cumulative scale, based on RKI/MZ data). $C_{0.5}$ and $C_{0.95}$ denote coverage rates of the 50% and 95% prediction intervals; AE and WIS stand for the mean absolute error and mean weighted interval score.

Germany

	3 wk ahead cumul case				4 wk ahead cumul case				3 wk ahead cumul death				4 wk ahead cumul death			
Model	AE	WIS	$C_{0.5}$	$C_{0.95}$	AE	WIS	$C_{0.5}$	$C_{0.95}$	AE	WIS	$C_{0.5}$	$C_{0.95}$	AE	WIS	$C_{0.5}$	$C_{0.95}$
epiforecasts-EpiExpert	56,806	36,975	0.33	0.58	90,856	59,030	0.25	0.58	1,409	930	0.50	0.92	2,325	1,515	0.33	0.92
epiforecasts-EpiNow2	93,015	68,352	0.58	0.67	168,222	121,460	0.58	0.67	2,070	1,401	0.50	0.67	3,244	2,189	0.42	0.75
FIAS_FZJ-Epi1Ger	72,325	45,809	0.17	0.92	122,165	79,579	0.08	0.58	1,792	1,290	0.08	0.50	2,774	1,946	0.08	0.58
Imperial-ensemble2																
itwm-dSEIR	56,386	42,720	0.33	0.42	101,463	75,670	0.33	0.42	1,677	1,459	0.25	0.42	2,265	1,945	0.17	0.50
ITWW-county_repro	94,211	75,775	0.00	0.42	160,665	128,935	0.00	0.42	826	658	0.00	0.17	740	589	0.42	0.67
Karlen-pypm	107,997	76,940	0.33	0.83	195,806	138,671	0.17	0.75	1,896	1,172	0.08	0.83	3,205	2,033	0.00	0.83
LANL-GrowthRate	44,978	38,134	0.75	1.00	72,617	57,154	0.67	1.00	1,557	963	0.42	1.00	2,447	1,572	0.42	1.00
LeipzigIMISE-SECIR	81,273	56,348	0.33	0.58	146,917	102,340	0.33	0.75	4,929	3,727	0.33	0.67	6,882	5,009	0.08	0.67
MIT_CovidAnalytics-DELPHI									2,152	1,404	0.17	0.67	2,608	1,759	0.08	0.67
SDSC_ISG-TrendModel																
USC-SIkJalpha	72,202	62,065	0.08	0.17	115,293	105,336	0.08	0.25	1,796	1,368	0.08	0.25	2,744	2,223	0.00	0.08
KIT-baseline	70,752	56,271	0.08	0.25	114,908	96,625	0.00	0.17	2,352	1,760	0.17	0.42	3,867	3,094	0.08	0.25
KIT-extrapolation_baseline	67,400	42,285	0.25	0.75	118,558	76,508	0.17	0.75	2,572	1,608	0.17	0.75	4,493	2,909	0.17	0.75
KIT-time_series_baseline	94,122	67,918	0.17	0.58	153,758	115,049	0.33	0.58	2,553	1,830	0.50	0.92	4,372	3,271	0.50	0.83
KITCOVIDhub-inverse_wis_ensemble	66,373	41,830	0.33	0.83	111,839	72,513	0.25	0.67	893	571	0.58	1.00	1,535	958	0.50	1.00
KITCOVIDhub-mean_ensemble	63,145	40,346	0.50	0.83	108,994	70,062	0.42	0.83	972	599	0.42	1.00	1,684	997	0.25	1.00
KITCOVIDhub-median_ensemble	58,244	38,895	0.42	0.75	100,388	68,015	0.42	0.83	1,104	641	0.33	1.00	1,854	1,072	0.33	1.00

Poland

	3 wk ahead cumul case				4 wk ahead cumul case				3 wk ahead cumul death				4 wk ahead cumul death			
Model	AE	WIS	$C_{0.5}$	$C_{0.95}$	AE	WIS	$C_{0.5}$	$C_{0.95}$	AE	WIS	$C_{0.5}$	$C_{0.95}$	AE	WIS	$C_{0.5}$	$C_{0.95}$
epiforecasts-EpiExpert	71,493	46,562	0.17	0.67	124,129	86,643	0.08	0.50	801	495	0.50	1.00	1,188	756	0.50	0.92
epiforecasts-EpiNow2	97,781	68,679	0.33	0.75	180,004	125,246	0.25	0.58	1,189	794	0.58	0.92	1,985	1,354	0.42	0.92
ICM-agentModel	* 67,819	* 49,027	0.55	1.00	*101,414	* 73,220	0.55	0.91	*1,207	*1,262	0.91	1.00	*1,834	*1,789	1.00	1.00
Imperial-ensemble2																
ITWW-county_repro	118,193	101,211	0.17	0.33	224,258	192,827	0.08	0.33	2,410	2,187	0.00	0.00	3,854	3,406	0.00	0.08
LANL-GrowthRate	71,765	42,183	0.50	1.00	127,178	74,690	0.08	0.83	1,027	616	0.33	0.92	1,638	977	0.33	0.83
MIMUW-StochSEIR	53,052	46,717	0.08	0.25	106,465	96,945	0.00	0.00	832	787	0.00	0.17	1,186	1,072	0.00	0.17
MIT_CovidAnalytics-DELPHI									*1,540	*1,075	0.18	0.55	*2,558	*1,978	0.09	0.36
MOCOS-agent1	38,471	32,124	0.42	0.58	73,460	59,498	0.42	0.50	531	422	1.00	1.00	699	589	0.92	1.00
SDSC_ISG-TrendModel																
USC-SIkJalpha	84,104	75,355	0.08	0.25	145,603	136,915	0.08	0.08	625	428	0.33	0.58	992	701	0.17	0.50
KIT-baseline	85,544	69,035	0.08	0.33	137,648	116,676	0.00	0.25	1,286	965	0.00	0.33	2,063	1,658	0.00	0.33
KIT-extrapolation_baseline	93,229	72,727	0.33	0.50	184,714	148,616	0.33	0.33	1,203	859	0.42	0.67	2,021	1,465	0.42	0.67
KIT-time_series_baseline	95,539	70,731	0.50	0.75	186,952	142,431	0.42	0.67	1,336	1,132	0.50	0.50	2,342	1,966	0.42	0.58
KITCOVIDhub-inverse_wis_ensemble	64,825	42,213	0.33	0.83	116,123	80,227	0.25	0.67	563	381	0.75	1.00	979	629	0.67	1.00
KITCOVIDhub-mean_ensemble	66,331	42,082	0.33	0.83	118,378	80,761	0.33	0.75	416	386	1.00	1.00	750	617	0.75	1.00
KITCOVIDhub-median_ensemble	68,709	46,381	0.42	0.83	120,285	85,310	0.25	0.50	382	332	0.75	1.00	667	565	0.67	1.00

*Asterisks mark entries where scores were imputed for at least one week. Weighted interval scores and absolute errors were imputed with the worst (largest) score achieved by any other forecast for the respective target and week. Models marked thus received a pessimistic assessment of their performance. If a model covered less than two thirds of the evaluation period, results are omitted.

Table S5: Forecast evaluation for Germany and Poland, 1 and 2 weeks ahead (incidence scale, based on JHU data). $C_{0.5}$ and $C_{0.95}$ denote coverage rates of the 50% and 95% prediction intervals; AE and WIS stand for the mean absolute error and mean weighted interval score.

	Germany															
	1 wk ahead case (JHU)				2 wk ahead case (JHU)				1 wk ahead death (JHU)				2 wk ahead death (JHU)			
Model	AE	WIS	$C_{0.5}$	$C_{0.95}$	AE	WIS	$C_{0.5}$	$C_{0.95}$	AE	WIS	$C_{0.5}$	$C_{0.95}$	AE	WIS	$C_{0.5}$	$C_{0.95}$
epiforecasts-EpiExpert	13,319	8,491	2/12	9/12	22,391	15,479	3/12	8/12	336	226	4/12	11/12	485	323	4/12	11/12
epiforecasts-EpiNow2	14,041	10,182	4/12	10/12	31,439	22,548	6/12	8/12	284	196	9/12	11/12	601	424	7/12	9/12
FIAS_FZJ-Epi1Ger	14,650	9,740	2/12	8/12	27,022	17,565	1/12	9/12	389	299	2/12	5/12	636	468	2/12	8/12
IHME-CurveFit									461				661			
Imperial-ensemble2									*234	*155	7/10	9/10				
itwm-dSEIR	9,919	7,887	6/12	7/12	20,214	14,003	5/12	9/12	492	319	6/12	11/12	518	336	6/12	10/12
ITWW-county_repro	17,888	15,162	2/12	4/12	33,039	27,132	1/12	3/12	517	483	1/12	2/12	259	204	1/12	2/12
Karlen-pypm	22,754	16,774	4/12	10/12	34,263	25,765	4/12	10/12	393	253	6/12	11/12	656	414	2/12	10/12
LANL-GrowthRate	12,175	10,877	9/12	12/12	17,749	14,958	9/12	11/12	286	193	6/12	12/12	457	286	6/12	12/12
LeipzigIMISE-SECIR	14,283	11,458	2/12	5/12	28,741	20,567	2/12	6/12	406	292	7/12	12/12	912	656	4/12	8/12
MIT_CovidAnalytics-DELPHI	*15,629	*10,544	5/11	10/11	*23,970	*16,970	3/11	8/11	777	468	5/12	12/12	763	452	7/12	12/12
SDSC_ISG-TrendModel	9,981								419							
USC-SIkJalpha	18,702	12,899	2/12	9/12	26,340	18,768	1/12	6/12	393	260	7/12	10/12	573	343	3/12	9/12
KIT-baseline	16,125	10,728	4/12	10/12	26,070	18,921	1/12	6/12	423	291	7/12	11/12	814	544	2/12	8/12
KIT-extrapolation_baseline	13,419	8,526	4/12	10/12	24,159	15,031	3/12	11/12	450	282	5/12	12/12	826	506	4/12	10/12
KIT-time_series_baseline	20,128	13,215	1/12	8/12	34,678	23,736	2/12	7/12	467	295	7/12	12/12	844	610	6/12	11/12
KITCOVIDhub-inverse_wis_ensemble	12,749	8,145	5/12	11/12	24,149	14,899	4/12	11/12	185	148	8/12	12/12	331	213	10/12	12/12
KITCOVIDhub-mean_ensemble	12,822	8,185	6/12	12/12	24,164	14,858	4/12	11/12	202	150	7/12	12/12	360	224	7/12	12/12
KITCOVIDhub-median_ensemble	11,789	7,853	3/12	10/12	21,667	14,075	4/12	10/12	195	147	8/12	12/12	381	231	7/12	12/12
	Poland															
	1 wk ahead case				2 wk ahead case				1 wk ahead death				2 wk ahead death			
Model	AE	WIS	$C_{0.5}$	$C_{0.95}$	AE	WIS	$C_{0.5}$	$C_{0.95}$	AE	WIS	$C_{0.5}$	$C_{0.95}$	AE	WIS	$C_{0.5}$	$C_{0.95}$
epiforecasts-EpiExpert	9,348	5,809	0.33	0.92	28,241	19,561	0.08	0.58	221	151	0.42	0.75	316	195	0.50	0.83
epiforecasts-EpiNow2	8,669	6,394	0.50	0.83	30,133	22,536	0.42	0.83	218	138	0.50	0.92	390	245	0.42	1.00
ICM-agentModel	*24,466	*16,579	0.27	0.91	*28,581	*19,023	0.73	1.00	*506	*296	0.73	1.00	*573	*504	0.82	1.00
IHME-CurveFit									352				472			
Imperial-ensemble2									*191	*143	0.30	0.60				
ITWW-county_repro	18,811	16,021	0.08	0.42	36,017	31,212	0.17	0.50	548	510	0.00	0.00	729	656	0.00	0.00
LANL-GrowthRate	10,738	6,547	0.67	1.00	26,194	16,557	0.42	0.92	249	146	0.08	0.83	378	236	0.25	0.83
MIMUW-StochSEIR	8,492	6,067	0.17	0.58	20,002	17,452	0.08	0.25	249	235	0.08	0.17	286	264	0.00	0.00
MIT_CovidAnalytics-DELPHI	*24,435	*14,346	0.20	0.90	*52,595	*35,605	0.10	0.40	*417	*260	0.45	0.91	*536	*314	0.27	1.00
MOCOS-agent1	8,086	6,272	0.50	0.58	17,320	13,263	0.25	0.50	159	134	0.67	1.00	159	141	0.83	1.00
SDSC_ISG-TrendModel	8,333								289							
USC-SIkJalpha	13,247	8,677	0.25	0.67	35,120	26,749	0.17	0.50	210	142	0.42	0.92	252	156	0.42	1.00
KIT-baseline	18,711	11,471	0.33	0.83	34,420	24,900	0.08	0.42	293	188	0.42	0.75	459	315	0.25	0.67
KIT-extrapolation_baseline	10,390	6,698	0.50	0.92	32,416	23,760	0.25	0.50	286	199	0.50	0.75	417	297	0.42	0.75
KIT-time_series_baseline	11,424	8,387	0.58	0.83	31,643	22,688	0.50	0.75	312	246	0.58	0.67	486	388	0.50	0.58
KITCOVIDhub-inverse_wis_ensemble	8,451	5,486	0.50	1.00	25,280	16,907	0.25	0.83	182	119	0.58	1.00	233	153	0.67	1.00
KITCOVIDhub-mean_ensemble	8,817	5,619	0.67	1.00	26,309	17,097	0.33	0.83	169	121	0.67	1.00	174	156	0.83	1.00
KITCOVIDhub-median_ensemble	8,977	5,411	0.50	0.92	25,951	17,939	0.17	0.75	190	114	0.58	1.00	215	142	0.75	1.00

*Asterisks mark entries where scores were imputed for at least one week. Weighted interval scores and absolute errors were imputed with the worst (largest) score achieved by any other forecast for the respective target and week. Models marked thus received a pessimistic assessment of their performance. If a model covered less than two thirds of the evaluation period, results are omitted.

Table S6: Forecast evaluation for Germany and Poland, 3 and 4 weeks ahead (incidence scale, based on JHU data). $C_{0.5}$ and $C_{0.95}$ denote coverage rates of the 50% and 95% prediction intervals; AE and WIS stand for the mean absolute error and mean weighted interval score.

	3 wk ahead case (JHU)				4 wk ahead case (JHU)				3 wk ahead death (JHU)				4 wk ahead death (JHU)			
**Germany** Model	AE	WIS	$C_{0.5}$	$C_{0.95}$	AE	WIS	$C_{0.5}$	$C_{0.95}$	AE	WIS	$C_{0.5}$	$C_{0.95}$	AE	WIS	$C_{0.5}$	$C_{0.95}$
epiforecasts-EpiExpert	30,920	20,680	3/12	7/12	38,187	24,940	2/12	8/12	724	470	3/12	11/12	971	646	2/12	9/12
epiforecasts-EpiNow2	51,165	35,835	5/12	9/12	74,985	54,568	6/12	8/12	946	659	6/12	9/12	1,260	866	6/12	9/12
FIAS_FZJ-Epi1Ger	37,482	25,141	1/12	6/12	52,812	36,746	1/12	7/12	871	592	1/12	7/12	1,071	678	1/12	6/12
IHME-CurveFit									874				1,000			
Imperial-ensemble2																
itwm-dSEIR	31,973	19,974	4/12	10/12	47,231	30,301	5/12	10/12	532	335	5/12	12/12	539	337	5/12	12/12
ITWW-county_repro	48,002	38,724	1/12	5/12	64,365	53,500	3/12	5/12	330	246	2/12	4/12	781	625	1/12	1/12
Karlen-pypm	55,203	39,877	3/12	8/12	91,822	66,228	2/12	8/12	952	628	0/12	10/12	1,342	939	0/12	7/12
LANL-GrowthRate	23,138	17,800	9/12	11/12	32,959	22,202	6/12	12/12	722	457	5/12	11/12	1,076	675	6/12	11/12
LeipzigIMISE-SECIR	48,868	33,487	1/12	8/12	77,302	54,290	3/12	8/12	1,589	1,102	3/12	8/12	2,526	1,798	2/12	7/12
MIT_CovidAnalytics-DELPHI	*29,198	*22,885	5/11	7/11	*39,778	*31,367	5/11	7/11	718	465	9/12	12/12	680	488	9/12	12/12
SDSC_ISG-TrendModel																
USC-SIkJalpha	35,113	26,616	2/12	6/12	49,985	39,839	1/12	2/12	815	519	3/12	8/12	948	619	2/12	7/12
KIT-baseline	37,102	28,457	1/12	4/12	46,124	37,251	0/12	3/12	1,188	858	2/12	6/12	1,529	1,159	1/12	3/12
KIT-extrapolation_baseline	36,004	23,750	2/12	9/12	54,153	37,117	2/12	6/12	1,351	866	2/12	9/12	1,917	1,302	2/12	9/12
KIT-time_series_baseline	46,542	34,947	4/12	7/12	63,585	47,872	3/12	6/12	1,318	981	6/12	10/12	1,815	1,452	5/12	10/12
KITCOVIDhub-inverse_wis_ensemble	35,449	21,286	3/12	11/12	49,251	31,504	5/12	9/12	505	320	9/12	12/12	717	473	8/12	12/12
KITCOVIDhub-mean_ensemble	35,296	21,200	2/12	10/12	48,995	31,027	5/12	10/12	537	319	8/12	12/12	745	454	8/12	12/12
KITCOVIDhub-median_ensemble	31,963	19,924	3/12	10/12	46,236	28,865	3/12	9/12	553	338	4/12	12/12	727	475	5/12	12/12
**Poland**	3 wk ahead case				4 wk ahead case				3 wk ahead death				4 wk ahead death			
Model	AE	WIS	$C_{0.5}$	$C_{0.95}$	AE	WIS	$C_{0.5}$	$C_{0.95}$	AE	WIS	$C_{0.5}$	$C_{0.95}$	AE	WIS	$C_{0.5}$	$C_{0.95}$
epiforecasts-EpiExpert	49,190	36,126	0.00	0.42	66,413	49,981	0.08	0.42	381	248	0.58	0.92	538	316	0.42	0.92
epiforecasts-EpiNow2	57,000	40,754	0.25	0.58	84,239	61,040	0.25	0.50	590	408	0.58	0.92	858	598	0.42	0.92
ICM-agentModel	*36,110	*21,706	0.45	0.91	* 43,759	*28,736	0.27	0.64	* 580	*533	0.91	1.00	* 656	* 544	0.82	1.00
IHME-CurveFit									603				521			
Imperial-ensemble2																
ITWW-county_repro	64,635	56,004	0.08	0.50	111,127	96,150	0.00	0.25	1,020	886	0.00	0.08	1,444	1,219	0.00	0.17
LANL-GrowthRate	45,833	27,090	0.17	0.75	58,322	37,589	0.17	0.75	480	300	0.50	0.83	640	395	0.58	0.83
MIMUW-StochSEIR	37,055	33,387	0.08	0.25	61,665	56,148	0.08	0.08	347	314	0.17	0.17	422	350	0.17	0.17
MIT_CovidAnalytics-DELPHI	*77,200	*55,508	0.10	0.40	* 96,731	*73,059	0.10	0.30	* 770	*476	0.45	0.91	*1,062	* 698	0.27	0.64
MOCOS-agent1	25,951	21,656	0.25	0.42	43,679	32,808	0.17	0.42	210	164	0.92	1.00	220	203	0.92	1.00
SDSC_ISG-TrendModel																
USC-SIkJalpha	50,909	42,933	0.08	0.25	66,710	59,344	0.08	0.33	244	160	0.58	1.00	408	270	0.42	0.92
KIT-baseline	44,230	36,646	0.17	0.33	56,963	48,028	0.08	0.25	647	481	0.08	0.50	812	631	0.00	0.50
KIT-extrapolation_baseline	57,609	47,303	0.33	0.42	102,746	82,981	0.17	0.33	576	431	0.50	0.67	899	667	0.33	0.58
KIT-time_series_baseline	56,074	43,716	0.42	0.67	97,935	75,620	0.33	0.67	701	585	0.33	0.58	1,058	861	0.17	0.50
KITCOVIDhub-inverse_wis_ensemble	41,559	29,313	0.17	0.67	62,808	45,573	0.25	0.58	268	190	0.75	1.00	444	280	0.50	1.00
KITCOVIDhub-mean_ensemble	44,185	30,162	0.25	0.58	66,277	46,910	0.25	0.50	228	197	0.75	1.00	403	283	0.75	1.00
KITCOVIDhub-median_ensemble	44,949	31,671	0.17	0.50	64,535	46,726	0.08	0.58	229	187	0.75	1.00	399	278	0.58	1.00

*Asterisks mark entries where scores were imputed for at least one week. Weighted interval scores and absolute errors were imputed with the worst (largest) score achieved by any other forecast for the respective target and week. Models marked thus received a pessimistic assessment of their performance. If a model covered less than two thirds of the evaluation period, results are omitted.

Table S7: Forecast evaluation at the regional level, Germany and Poland, 3 and 4 weeks ahead (incidence scale, based on RKI/MZ data). Results are averaged over the different regions (states in Germany, voivodeships in Poland). $C_{0.5}$ and $C_{0.95}$ denote coverage rates of the 50% and 95% prediction intervals; AE and WIS stand for the mean absolute error and mean weighted interval score.

	**Germany**															
	3 wk ahead case				4 wk ahead case				3 wk ahead death				4 wk ahead death			
Model	AE	WIS	$C_{0.5}$	$C_{0.95}$	AE	WIS	$C_{0.5}$	$C_{0.95}$	AE	WIS	$C_{0.5}$	$C_{0.95}$	AE	WIS	$C_{0.5}$	$C_{0.95}$
epiforecasts-EpiNow2	2,697	1,803	0.33	0.73	3,879	2,630	0.29	0.70	98	66	0.44	0.80	136	95	0.42	0.78
FIAS_FZJ-Epi1Ger	2,840	1,929	0.19	0.58	4,192	2,862	0.18	0.52	75	54	0.16	0.45	96	65	0.13	0.51
IHME-CurveFit									64				68			
ITWW-county_repro	3,123	2,100	0.39	0.77	4,394	2,970	0.44	0.78	34	23	0.34	0.79	52	34	0.31	0.77
Karlen-pypm	3,634	2,261	0.43	0.84	5,740	3,578	0.30	0.75	64	36	0.41	0.92	84	49	0.33	0.85
LeipzigIMISE-SECIR			0.17	0.50			0.17	0.67			0.08	0.33			0.17	0.42
USC-SIkJalpha	2,190	1,584	0.23	0.65	2,830	2,094	0.19	0.54	53	36	0.35	0.82	65	45	0.30	0.81
KIT-baseline	2,194	1,636	0.17	0.50	2,774	2,168	0.11	0.40	79	54	0.25	0.69	96	69	0.20	0.57
KIT-extrapolation_baseline	2,496	1,629	0.21	0.69	3,392	2,315	0.16	0.60	114	76	0.36	0.78	157	106	0.35	0.75
KIT-time_series_baseline	2,806	1,989	0.20	0.61	3,571	2,748	0.21	0.55	132	90	0.38	0.78	178	130	0.32	0.73
KITCOVIDhub-inverse_wis_ensemble	2,622	1,675	0.38	0.82	3,679	2,375	0.35	0.79	55	35	0.41	0.93	77	51	0.39	0.89
KITCOVIDhub-mean_ensemble	2,503	1,582	0.37	0.86	3,496	2,254	0.37	0.80	61	39	0.44	0.93	86	58	0.44	0.89
KITCOVIDhub-median_ensemble	2,589	1,607	0.32	0.85	3,669	2,323	0.27	0.76	51	32	0.48	0.95	70	45	0.40	0.92
	**Poland**															
	3 wk ahead case				4 wk ahead case				3 wk ahead death				4 wk ahead death			
Model	AE	WIS	$C_{0.5}$	$C_{0.95}$	AE	WIS	$C_{0.5}$	$C_{0.95}$	AE	WIS	$C_{0.5}$	$C_{0.95}$	AE	WIS	$C_{0.5}$	$C_{0.95}$
epiforecasts-EpiNow2	3,849	2,745	0.36	0.69	5,889	4,209	0.27	0.61	77	63	0.45	0.83	149	194	0.41	0.83
ITWW-county_repro	4,319	3,222	0.37	0.73	7,133	5,353	0.29	0.66	71	53	0.20	0.60	98	70	0.26	0.69
USC-SIkJalpha	2,543	1,910	0.24	0.54	3,438	2,665	0.21	0.49	28	18	0.48	0.91	35	23	0.44	0.88
KIT-baseline	2,743	2,195	0.16	0.40	3,519	2,932	0.10	0.34	47	32	0.30	0.70	58	43	0.27	0.61
KIT-extrapolation_baseline	4,002	3,073	0.26	0.57	7,041	5,491	0.20	0.48	52	34	0.44	0.86	70	48	0.39	0.83
KIT-time_series_baseline	3,905	2,855	0.34	0.66	6,563	5,056	0.28	0.60	58	43	0.31	0.59	75	57	0.23	0.58
KITCOVIDhub-inverse_wis_ensemble	3,327	2,253	0.32	0.73	5,558	3,892	0.23	0.65	32	22	0.54	0.94	48	46	0.51	0.93
KITCOVIDhub-mean_ensemble	3,107	2,051	0.33	0.80	5,205	3,560	0.23	0.67	34	25	0.55	0.94	59	61	0.52	0.93
KITCOVIDhub-median_ensemble	3,301	2,095	0.35	0.76	5,412	3,441	0.24	0.62	30	20	0.54	0.92	40	28	0.51	0.89

*Asterisks mark entries where scores were imputed for at least one week. Weighted interval scores and absolute errors were imputed with the worst (largest) score achieved by any other forecast for the respective target and week. Models marked thus received a pessimistic assessment of their performance. If a model covered less than two thirds of the evaluation period, results are omitted.

Table S8: Forecast evaluation for Germany and Poland, pooled across evaluation periods, 1 and 2 weeks ahead (incidence scale, based on RKI/MZ data). $C_{0.5}$ and $C_{0.95}$ denote coverage rates of the 50% and 95% prediction intervals; AE and WIS stand for the mean absolute error and mean weighted interval score.

**Germany**	1 wk ahead case				2 wk ahead case				1 wk ahead death				2 wk ahead death			
Model	AE	WIS	$C_{0.5}$	$C_{0.95}$	AE	WIS	$C_{0.5}$	$C_{0.95}$	AE	WIS	$C_{0.5}$	$C_{0.95}$	AE	WIS	$C_{0.5}$	$C_{0.95}$
epiforecasts-EpiExpert	10,653	6,945	0.36	0.86	24,560	17,271	0.33	0.43	249	171	0.50	0.82	433	285	0.33	0.81
epiforecasts-EpiNow2	10,355	7,229	0.59	0.77	32,772	23,970	0.52	0.71	246	157	0.64	0.82	493	339	0.52	0.76
FIAS_FZJ-Epi1Ger	9,118	6,028	0.50	0.91	27,174	18,523	0.29	0.67	354	285	0.23	0.32	599	457	0.19	0.48
IHME-CurveFit																
Imperial-ensemble2									*224	*166	0.65	0.70				
itwm-dSEIR			0.42	0.75			0.42	0.75			0.58	0.83			0.50	0.83
ITWW-county_repro	23,951	19,913	0.05	0.23	45,782	37,544	0.00	0.19	476	449	0.05	0.09	393	342	0.10	0.24
Karlen-pypm			0.43	0.93			0.23	0.85			0.36	0.86			0.08	0.77
LANL-GrowthRate	*22,330	*15,271	0.74	1.00	*36,344	*23,394	0.61	1.00	*285	*187	0.47	1.00	*436	*281	0.50	0.94
LeipzigIMISE-SECIR	14,097	*13,366	0.24	0.65	37,135	*36,634	0.12	0.50	484	*318	0.41	0.76	828	*621	0.31	0.62
MIT_CovidAnalytics-DELPHI	*24,290	*17,004	0.37	0.74	*44,566	*33,677	0.33	0.56	*671	*436	0.30	0.70	*636	*397	0.37	0.89
SDSC_ISG-TrendModel	9,271								400							
UCLA-SuEIR																
USC-SIkJalpha	16,613		0.31	0.85	27,942		0.17	0.58	430		0.46	0.77	582		0.25	0.83
KIT-baseline	15,355	10,246	0.45	0.91	27,601	20,850	0.24	0.62	442	270	0.41	0.91	804	518	0.10	0.62
KIT-extrapolation_baseline	10,274	7,900	0.59	1.00	28,704	19,614	0.48	0.76	341	208	0.50	0.95	624	385	0.43	0.86
KIT-time_series_baseline	15,492	10,614	0.36	0.82	37,524	24,854	0.33	0.76	330	230	0.64	0.95	754	521	0.48	0.90
KITCOVIDhub-inverse_wis_ensemble	11,055	7,141	0.55	0.95	30,599	19,896	0.38	0.71	200	133	0.59	0.95	285	181	0.52	0.95
KITCOVIDhub-mean_ensemble	12,137	7,732	0.59	0.91	30,563	19,502	0.33	0.81	213	146	0.45	0.95	326	199	0.52	0.95
KITCOVIDhub-median_ensemble	9,249	6,221	0.59	0.95	27,149	17,998	0.38	0.81	217	143	0.59	0.91	358	221	0.48	0.90

**Poland**	1 wk ahead case				2 wk ahead case				1 wk ahead death				2 wk ahead death			
Model	AE	WIS	$C_{0.5}$	$C_{0.95}$	AE	WIS	$C_{0.5}$	$C_{0.95}$	AE	WIS	$C_{0.5}$	$C_{0.95}$	AE	WIS	$C_{0.5}$	$C_{0.95}$
epiforecasts-EpiExpert	10,292	6,836	0.59	0.86	30,493	20,653	0.19	0.62	243	155	0.36	0.95	423	264	0.33	0.86
epiforecasts-EpiNow2	9,327	6,422	0.55	0.82	33,681	23,474	0.33	0.76	276	184	0.45	0.86	670	465	0.43	0.76
ICM-agentModel	*19,736	*13,900	0.33	0.93	*32,194	*21,406	0.57	0.86	*599	*453	0.53	0.74	*1,101	*791	0.50	0.72
IHME-CurveFit																
Imperial-ensemble2									*293	*188	0.30	0.70				
ITWW-county_repro	19,188	16,147	0.18	0.32	35,214	29,629	0.19	0.48	560	521	0.00	0.05	748	669	0.00	0.10
LANL-GrowthRate	*11,012	* 7,151	0.68	1.00	*31,944	*19,233	0.44	0.94	*233	*151	0.32	0.89	* 366	*228	0.39	0.94
MIMUW-StochSEIR	*10,072	* 6,468	0.41	0.88	*23,492	*19,354	0.25	0.38	*422	*320	0.18	0.35	* 771	*557	0.06	0.12
MIT_CovidAnalytics-DELPHI	*27,212	*17,817	0.21	0.74	*54,488	*38,714	0.11	0.61	*447	*282	0.40	0.80	* 580	*354	0.26	0.89
MOCOS-agent1	8,855	6,863	0.32	0.59	22,131	17,207	0.14	0.52	174	139	0.82	1.00	296	202	0.81	0.95
SDSC_ISG-TrendModel	6,918								214							
USC-SIkJalpha	10,353		0.31	0.85	29,100		0.17	0.50	206		0.31	0.92	255		0.42	0.92
KIT-baseline	21,751	13,546	0.45	0.86	41,057	28,022	0.14	0.52	340	216	0.45	0.95	595	384	0.19	0.71
KIT-extrapolation_baseline	13,477	8,685	0.55	0.95	40,533	27,328	0.29	0.67	332	233	0.59	0.82	658	463	0.48	0.81
KIT-time_series_baseline	16,108	10,657	0.64	0.91	43,096	28,569	0.52	0.86	411	281	0.64	0.82	854	574	0.52	0.71
KITCOVIDhub-inverse_wis_ensemble	9,796	6,402	0.45	0.95	28,909	19,458	0.38	0.76	182	130	0.68	1.00	321	216	0.62	0.95
KITCOVIDhub-mean_ensemble	9,646	6,391	0.55	0.95	29,101	19,012	0.33	0.76	191	136	0.73	0.95	349	242	0.71	0.95
KITCOVIDhub-median_ensemble	10,342	6,430	0.55	0.95	30,228	19,832	0.29	0.76	186	124	0.64	1.00	312	202	0.52	1.00

*Asterisks mark entries where scores were imputed for at least one week. Weighted interval scores and absolute errors were imputed with the worst (largest) score achieved by any other forecast for the respective target and week. Models marked thus received a pessimistic assessment of their performance. If a model covered less than two thirds of the evaluation period, results are omitted.

Table S9: Forecast evaluation for Germany and Poland, pooled across evaluation periods, 3 and 4 weeks ahead (incidence scale, based on RKI/MZ data). $C_{0.5}$ and $C_{0.95}$ denote coverage rates of the 50% and 95% prediction intervals; AE and WIS stand for the mean absolute error and mean weighted interval score.

Germany

Model	3 wk ahead case AE	WIS	$C_{0.5}$	$C_{0.95}$	4 wk ahead case AE	WIS	$C_{0.5}$	$C_{0.95}$	3 wk ahead death AE	WIS	$C_{0.5}$	$C_{0.95}$	4 wk ahead death AE	WIS	$C_{0.5}$	$C_{0.95}$
epiforecasts-EpiExpert	36,332	25,966	0.25	0.55	48,205	32,980	0.05	0.63	673	449	0.25	0.70	936	677	0.21	0.63
epiforecasts-EpiNow2	70,236	51,689	0.40	0.75	134,886	98,969	0.37	0.63	826	574	0.45	0.75	1,336	906	0.42	0.74
FIAS_FZJ-Epi1Ger	53,281	37,314	0.15	0.65	88,229	64,186	0.16	0.63	877	648	0.05	0.50	1,046	737	0.16	0.53
IHME-CurveFit																
Imperial-ensemble2																
itwm-dSEIR			0.33	0.83			0.42	0.92			0.42	0.92			0.42	1.00
ITWW-county_repro	70,730	58,490	0.05	0.30	96,380	81,756	0.05	0.32	495	405	0.15	0.35	770	625	0.11	0.21
Karlen-pypm			0.33	0.75			0.17	0.67			0.00	0.75			0.00	0.67
LANL-GrowthRate	*45,698	*29,603	0.53	0.94	* 45,825	* 33,757	0.56	0.94	* 751	* 502	0.47	0.88	*1,044	* 711	0.50	0.88
LeipzigIMISE-SECIR	62,495	*72,724	0.07	0.53	102,043	*127,685	0.07	0.57	1,295	*1,108	0.20	0.60	2,003	*1,946	0.21	0.57
MIT_CovidAnalytics-DELPHI	*71,468	*58,057	0.28	0.56	*104,826	* 87,522	0.28	0.44	* 669	* 465	0.53	0.84	817	636	0.53	0.74
SDSC_ISG-TrendModel																
UCLA-SuEIR																
USC-SIkJalpha	41,039		0.08	0.42	53,915		0.17	0.25	808		0.25	0.67	1,007		0.25	0.67
KIT-baseline	38,805	30,289	0.25	0.45	48,062	37,794	0.16	0.37	1,188	842	0.10	0.40	1,536	1,186	0.05	0.21
KIT-extrapolation_baseline	54,987	36,555	0.30	0.85	93,240	64,624	0.21	0.58	1,099	711	0.30	0.80	1,758	1,169	0.16	0.79
KIT-time_series_baseline	65,037	45,753	0.30	0.70	98,051	78,394	0.37	0.68	1,216	905	0.40	0.75	1,762	1,545	0.37	0.79
KITCOVIDhub-inverse_wis_ensemble	56,618	38,957	0.25	0.75	94,005	65,809	0.32	0.74	473	301	0.65	0.95	687	458	0.47	0.89
KITCOVIDhub-mean_ensemble	53,170	35,696	0.20	0.70	81,675	57,091	0.32	0.74	506	310	0.45	0.95	699	434	0.42	0.95
KITCOVIDhub-median_ensemble	49,788	33,166	0.30	0.85	76,681	51,457	0.26	0.68	523	333	0.40	0.90	657	452	0.47	0.89

Poland

Model	3 wk ahead case AE	WIS	$C_{0.5}$	$C_{0.95}$	4 wk ahead case AE	WIS	$C_{0.5}$	$C_{0.95}$	3 wk ahead death AE	WIS	$C_{0.5}$	$C_{0.95}$	4 wk ahead death AE	WIS	$C_{0.5}$	$C_{0.95}$
epiforecasts-EpiExpert	55,346	40,055	0.05	0.30	81,957	62,080	0.05	0.26	571	369	0.40	0.80	800	520	0.32	0.74
epiforecasts-EpiNow2	73,258	51,543	0.25	0.65	146,975	104,951	0.26	0.42	1,388	970	0.35	0.75	2,471	1,748	0.32	0.79
ICM-agentModel			0.46	0.77			0.33	0.58	*1,037	* 854	0.65	0.76	*1,020	* 846	0.62	0.81
IHME-CurveFit																
Imperial-ensemble2																
ITWW-county_repro	65,634	56,584	0.10	0.45	122,956	107,847	0.05	0.26	1,073	932	0.10	0.15	1,669	1,446	0.05	0.11
LANL-GrowthRate	*57,040	*31,430	0.12	0.88	* 72,916	* 45,076	0.12	0.75	* 530	* 329	0.41	0.94	* 774	* 479	0.56	0.88
MIMUW-StochSEIR	*42,959	*37,974	0.13	0.27	* 70,955	* 62,821	0.07	0.07	*1,403	*1,004	0.07	0.13	*2,131	*1,468	0.07	0.29
MIT_CovidAnalytics-DELPHI	*90,391	*67,331	0.12	0.41	*125,485	*100,228	0.19	0.31	* 875	* 589	0.28	0.83	*1,313	* 955	0.24	0.65
MOCOS-agent1	41,792	35,988	0.25	0.40	76,914	65,186	0.26	0.37	517	333	0.75	0.85	847	592	0.63	0.79
SDSC_ISG-TrendModel																
USC-SIkJalpha	42,643		0.08	0.33	51,952		0.08	0.33	344		0.50	1.00	530		0.42	0.92
KIT-baseline	55,156	42,440	0.10	0.40	67,121	52,517	0.16	0.37	854	615	0.15	0.50	1,068	828	0.11	0.47
KIT-extrapolation_baseline	83,345	62,296	0.35	0.55	165,052	127,853	0.21	0.37	1,116	845	0.60	0.70	2,046	1,585	0.47	0.58
KIT-time_series_baseline	81,657	61,117	0.50	0.80	144,083	125,616	0.47	0.79	1,280	918	0.45	0.70	1,777	1,322	0.37	0.68
KITCOVIDhub-inverse_wis_ensemble	54,734	39,215	0.20	0.60	96,497	74,679	0.26	0.53	560	366	0.55	0.90	1,070	701	0.42	0.89
KITCOVIDhub-mean_ensemble	56,238	39,027	0.25	0.65	97,337	73,756	0.26	0.53	567	395	0.55	0.90	1,045	705	0.47	0.89
KITCOVIDhub-median_ensemble	54,928	40,367	0.20	0.55	92,043	70,201	0.05	0.53	476	317	0.60	0.95	913	589	0.47	0.84

*Asterisks mark entries where scores were imputed for at least one week. Weighted interval scores and absolute errors were imputed with the worst (largest) score achieved by any other forecast for the respective target and week. Models marked thus received a pessimistic assessment of their performance. If a model covered less than two thirds of the evaluation period, results are omitted.
