# Supplementary material for: National and subnational short-term forecasting of COVID-19 in Germany and Poland during early 2021
Source: Commun Med (Lond). 2022 Oct 31;2:136. doi: 10.1038/s43856-022-00191-8 (PMC9622804; doi:10.1038/s43856-022-00191-8)
Supplement: Supplementary file 5 — Description of Additional Supplementary Files [file 43856_2022_191_MOESM5_ESM.pdf]

Description of Additional Supplementary Files

File name: Supplementary Data 1

Description:

This supplementary data item contains all data required to reproduce Figures 1-7 of the main manuscript. It consists of a zip archive containing several csv files. The different files correspond to the different figures as follows:

- Data shown in Figure 1 is contained in the following files:
  - (a) incident cases: inc_case.csv
  - (b) incident deaths: inc_death.csv
  - (c) proportion of B117: variants.csv
  - (d) proportion of positive tests: positivity.csv
  - (e) stringency index: stringency.csv
  - (f) percentage vaccinated: vaccination.csv

- Data shown in Figures 2, 3, 5, 6, 7 are available in forecasts.csv.

  Columns:
  - model: the model
  - forecast_date: the date the forecast was issued
  - location: the location to which the forecast refers
  - target: the forecasting target (case or death, horizon)
  - value.point: the point prediction, usually the same as value.0.5
  - value.0.025, ..., value.0.975: the different predictive quantiles shown in the figures

- Data shown in Figure 4 are available in scores.csv.

  Columns:
  - model: the model
  - target: the forecasting target (case or death)
  - location: the location to which the forecast refers
  - ae.1, ..., ae.4: mean absolute errors at horizons 1 through 4 weeks
  - wis.1, ..., wis.4: mean weighted interval scores at horizons 1 through 4 weeks
